# Non-thermal plasma directly accelerates neuronal proliferation by stimulating axon formation

**DOI:** 10.1038/s41598-022-20063-4

**Published:** 2022-09-23

**Authors:** Chun Byung Do, M. Shriya Jaiswal, Yoon-Seo Jang, Uk-Kyu Kim, Gyoo-Cheon Kim, Dae-Seok Hwang

**Affiliations:** 1 Department of Oral and Maxillofacial Surgery, Dental and Life Science Institute, Dental School, Pusan National University, Busan, South Korea; 2 Deptartment of Oral Anatomy, School of Dentistry, Pusan National University, Yangsan, 50612 South Korea; 3 Dental Research Institute, Pusan National University Dental Hospital, Yangsan, South Korea

**Keywords:** Molecular biology, Neuroscience, Medical research, Molecular medicine

## Abstract

Among the various methods, Non Thermal Plasma (NTP) has been recently introduced and is being studied to recover the damaged nerve. In the recent years, several studies have suggested that NTP accelerates nerve cell regeneration, but the mechanism remains unknown. This study evaluated the effect of NTP on neuronal proliferation in SH-SY5Y (Human neuroblastoma cells) cells differentiated by retinoic acid (RA) and investigated the mechanism by which NTP promotes cell proliferation. We analyzed the morphology of differentiated SH-SY5Y cells, and performed western blot analysis and reverse transcription polymerase chain reaction (RT-PCR). Immunofluorescence analysis was performed in an in vivo study by categorizing Wistar A rats into three groups: non-nerve damage (Non-ND), nerve damage (ND), and nerve damage + NTP treatment (ND + NTP). The cell morphology analysis revealed that the number of cells increased and axonal elongation progressed after NTP treatment. In addition, western blots indicated that tau expression increased significantly after NTP treatment. The RT-PCR results revealed that the expression of tau, wnt3a, and β-catenin increased after NTP treatment. The in vivo immunofluorescence assay showed that NTP increased the markers for tau and S100B while regulating the over-expression of MAP2 and GAP43. NTP treatment accelerated cell proliferation and regeneration of damaged neurons in differentiated SH-SY5Y cells. These results establish the fact of NTP as a noninvasive and effective treatment for nerve injury.

## Introduction

Peripheral nerve injury occurs frequently due to trauma^1^, neurodegenerative diseases, such as diabetes^2^, Guillain–Barre syndrome, and carpal tunnel syndrome^3^, or iatrogenic causes, such as implant invasion of the mandibular canal^4^. Unlike other tissues, nerve recovery after injury is slow and often incomplete. Therefore, several methods of nerve regeneration are being studied, including surgery, medication, and gene therapy; however, these methods are overall ineffective.

Plasma can be created artificially by heating a neutral gas or exposing it to a strong electromagnetic field. They can be classified into two groups, thermal and non-thermal, according to its temperature^5^. Non-thermal plasma (NTP) is created by applying an electric or electromagnetic field to a gas^6^.

Plasma medicine is a new and growing field that combines clinical medicine, plasma physics, and life sciences. Since the first clinical trials in 2010 that used NTP to reduce bacteria in chronic wounds^7^, many experiments have revealed that it produces anti-cancer^8^, muscle regeneration^9^, wound healing^10^, and anti-inflammatory effects^11^. Usually, NTP is used directly in medical therapies or indirectly for processing surfaces, materials, or devices for medical applications^12^.

Initially, most of the effort in realizing the potential to apply plasma directly for medical purposes was in the field of dermatology, including wound healing. However, recently, treatments for cancer, nerve regeneration, and dentistry using NTP have been studied.

In the field of nerve regeneration, a few studies have suggested that the use of NTP promotes the regeneration of nerve cells. Lee et al.^13^ reported the accelerated recovery of damaged nerves when NTP was applied to the rat model of sciatic nerve crush injury. Other studies have also confirmed that NTP is helpful in nerve regeneration, but the exact mechanism of its activity remains unknown.

This study aimed to evaluate the effects of NTP on neuronal proliferation and investigate the mechanism by which NTP accelerates proliferation through in vitro and in vivo experiments.

## Materials and methods

### NTP device

**Figure 1:**
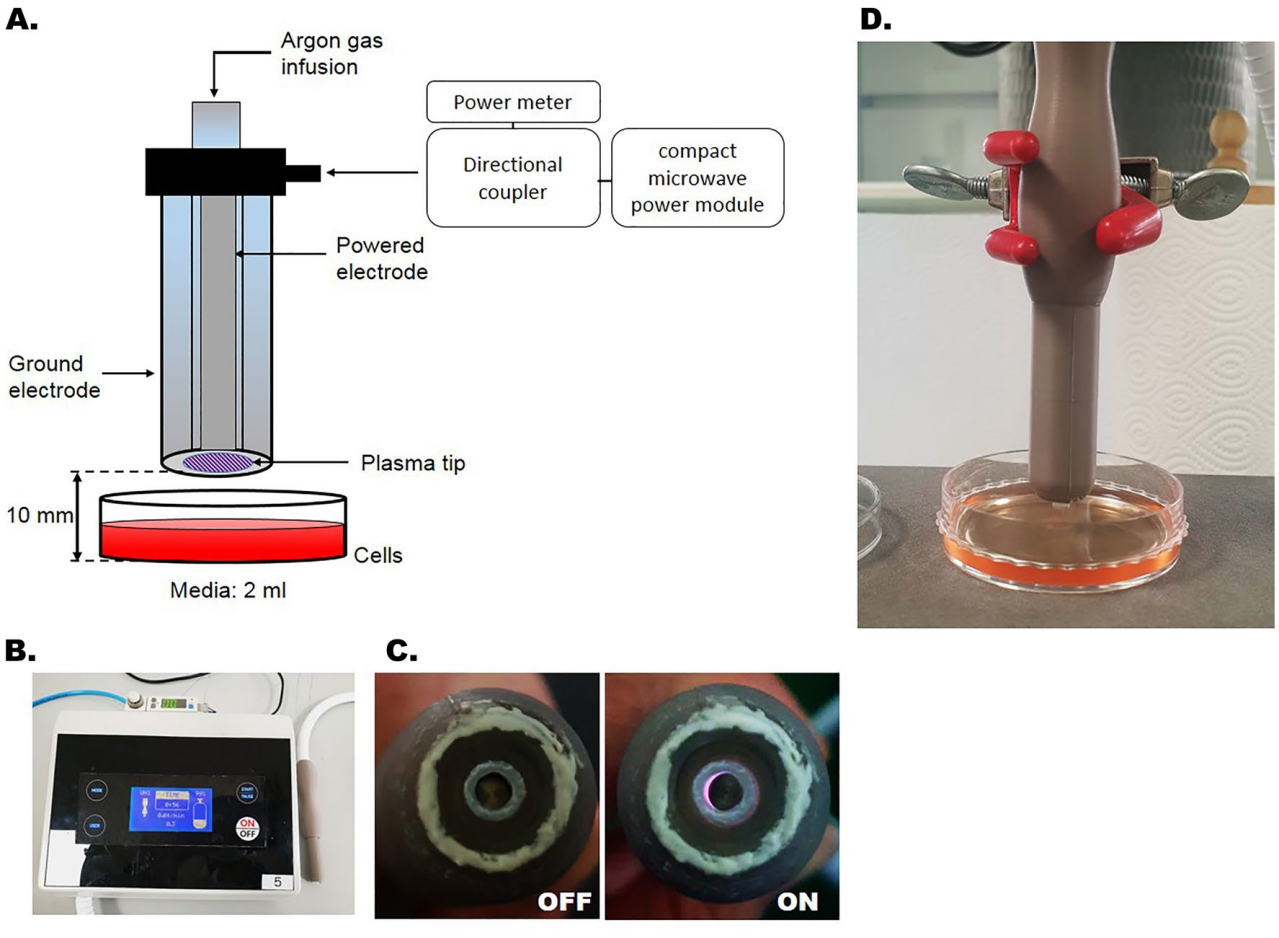
The NTP-generating device. (**A**) Principle sketch of NTP device and its generation. (**B**) NTP generated from the device. (**C**) Plasma glow within the electrodes. (**D**) NTP treatment of cells. The electrodes were maintained at a distance of 1 cm from the dish.

A dielectric-barrier-discharge-type NTP device developed by the FEAGLE Corporation (Yangsan-si, South Korea) was adopted for this study. Argon gas was used as the buffer gas and blown into the plasma source at 2.0 standard liters per minute (slm). NTP was formed by applying a high voltage (3 kV) to the plasma source. Plasma glow was formed within the electrodes, but it did not extend to the end of the electrodes. At a point 1 cm from the electrode tip, the temperature of the NTP flow was maintained under 35 °C for 10 min, and no UV radiation was detected. The distance of the dish from the electrodes was maintained at 1 cm (Fig. 1). In all experiments, treatment with NTP was applied at 2.0 slm for 5 min^14–16^.

### Cell culture

SH-SY5Y cells (ATCC, no. CRL-2266) were cultured in Dulbecco’s modified Eagle’s medium F-12 (HyClone, USA) supplemented with 10% fetal bovine serum (Hyclone, USA) at 37 °C in a humidified atmosphere containing 5% CO_2_. Cells were incubated for 48 h, then cells were treated with retinoic acid (RA, cat. R2625, Sigma-Aldrich, Bornem, Belgium) at 10 μM for 7 days to induce differentiation.

### Cell morphology analysis

An optical microscope (CX31, Olympus, and Tokyo, Japan) was used to confirm the differences in cell morphology. Images were captured using an iCM 9.0 digital camera system (IMT I-solution Inc., NY, USA).

### Western blot

Following 7 days of differentiation, cells were treated with NTP at 2.0 slm for 5 min and incubated for 0, 1, 3, 6, 24, and 48 h. The cells were washed with ice-cold phosphate-buffered saline (PBS) and lysed using radio immunoprecipitation assay lysis buffer (1 ml per 107 cells/100 mm dish/150 cm^2^ flask; 0.5 ml per 5 × 106 cells/60 mm dish/75 cm^2^ flask). After holding at 4 °C for 30 min, the tubes were centrifuged for 20 min at 12,000 rpm and 4 °C. Supernatants were isolated and transferred to new tubes. The total protein content of the lysate was quantified using a Bio-Rad protein assay (Bio-Rad Laboratories, Hercules, CA). The lysate samples (25–30 μg) were mixed with 5× sample buffer and then boiled at 95 °C for 5 min. Subsequently, each sample was separated by electrophoresis using 8–15% polyacrylamide SDS gel. The separated gel was transferred to PVDF membrane (Merck, NJ, USA) again. After the transfer, the protein markers marked on the membrane were cut according to the size of each antibody. Ultimately, the membranes were probed and analyzed using the following primary antibodies: MAP2 (1:200; Santa Cruz Biotechnology), tau (1:200; ABCAM), and GAP43 (1:10,000; ABCAM). The specific bands were detected with advanced enhanced chemiluminescence (ECL) western blotting reagents (Merck Millipore, Darmstadt, Germany). An anti-GAPDH antibody was used as a loading control (Santa Cruz Biotechnology). The membranes were incubated overnight with the primary and secondary antibodies at 4 °C, treated with ECL western blotting reagent and captured using an ImageQuant LAS 4000 (GE, Piscataway, NJ, USA).

### Reverse transcription polymerase chain reaction (RT-PCR) analysis

Following 7 days of differentiation, cells were treated with NTP at 2.0 slm; for 5 min and incubated for 0, 6, 16, and 24 h. The cells were washed with ice-cold PBS, and the total RNA was extracted using TRIzolTM reagent (Thermo Fisher Scientific), as described in the manufacturer’s instructions. First strand cDNA was synthesized using an RNA template and the specific primers, as described in the One-step PreMix kit (iNtRON Biotechnology Inc., Korea). Then, the same amounts of cDNA were then amplified via PCR in a 20 μl reaction volume containing 2× PCR master mix (Doctor Protein Corp., Korea).

This cDNA was used for PCR against tau, wnt3a, glycogen synthase kinase 3β (GSK3β), β-catenin and β-actin. The PCR primer sequences were as follows: tau, 5′-CAACTCAAAGCTCGCATGGT-3′ and 5′-TCCCCTGATTTTGGAGGTTC-3′; wnt3a, 5′-CTGGAGCTAGTGTCTCCTCTC-3′ and 5′-CCACCATAAAACCCCACTCC-3′; GSK3β, 5′-ATGTATGGTCTGCTGGCTGT-3′ and 5′-GAATCCGAGCATGAGGAGGA-3′; and β-actin, 5′-TCTGAGGAGCAGCTTCAGTC-3′ and 5′-ACATAGCAGCTCGTACCCTC-3′. The amplification products were separated by 1.8% agarose gel electrophoresis and visualized using ethidium bromide staining under UV trans illumination.

### Immunofluorescence assay (in vitro)

Following 7 days of differentiation, cells were treated with NTP at 2.0 slm; for 5 min and incubated for 24 h. Cells were fixed with 4% paraformaldehyde. Cells were then blocked with 0.5% Tween 20/10 BSA solution for 1 h at room temperature; and treated overnight with the primary antibodies, tau and β-tubulin at 4 °C. Then, they were washed three times with PBS for 5 min each; and incubated for 1 h at room temperature using Alexa 488-conjugated goat anti-mouse IgG antibody; and Alexa 594-conjugated goat anti-rabbit IgG antibody; (Invitrogen, Carlsbad, CA, USA, 1:200). The expression of tau and β-tubulin was detected using a confocal microscope.

### Animal experiments

A total of 10 male Wistar A rats weighing 250–300 g, 8–10 weeks old, were obtained from Samtako Bio (Osan-si, Gyeonggi-do, Republic of Korea). All experiments complied with the ethical guidelines of the Pusan National University Institutional Animal Care and Use Committee (PNUIACUC, South Korea) and regulations set by the International Association for the Study of Pain in Animals (ED-PNU 2020-2668). The experimental protocols were approved by Pusan National University Institutional Animal Care and Use Committee, South Korea. This study was done in accordance with ARRIVE guidelines.

The experiment was conducted in three groups according to the applied NTP. The surgical procedure was performed aseptically. A skin incision 2 cm in length was made parallel to the femur, and the subcutaneous tissue and femoral muscle layers were dissected carefully. A custom retractor was immobilized to isolate the right sciatic nerve and maintain exposure of the sciatic nerve in the operating field. In group 1 (non-nerve damage, non-ND), the sciatic nerve was surgically isolated but not cut as a sham operation. In groups 2 (nerve damage, ND) and 3 (nerve damage and NTP application, ND + NTP), the sciatic nerve was isolated and cut 5 mm proximal to the sciatic trifurcation. The damaged nerve was then immediately anastomosed to the epineurium with 8-0 monofilament suture. The muscle layer was closed with absorbable suture, and the skin layer was sutured with non-absorbable monofilament suture. Then, group 3 received NTP to the skin over the injured sciatic nerve three times a week for 8 weeks (Fig. 6A).

All rats were sacrificed 1 day after their last NTP treatment. Specimens were taken from the sciatic nerve in the field of surgery. For immunofluorescence analysis, the 5um nerve tissue sections were treated with antibodies against MAP2 (1:100, Santa Cruz Biotechnology/sc-74421, Santa Cruz, CA) and GAP-43 (1:500, ABCAM/ab75810, Cambridge, United Kingdom) for 2 h at 37 °C. Then, Samples were washed four times with PBS and treated with anti-mouse Alexa Fluor-488 and anti-rabbit Alexa Fluor-594 dyes (1:100, Thermo Fisher Scientific, Rockford, IL) at 37 °C for 1 h. Cell nuclei were then counterstained with 4′,6-diamidino-2-phenylindole. All tissue sections were also treated with antibodies against tau (1:200, ABCAM/ab80579, Cambridge, United Kingdom) and S100 calcium-binding protein B (S100B) (1:100, ABCAM/ab868, Cambridge, United Kingdom). Immunofluorescence images were obtained using a Carl Zeiss LSM 780 confocal laser microscope.

### Statistical analysis

Each experiment was repeated three times with standard deviations indicated as error bars. Experimental and control groups were compared using Excel’s paired t-test (Microsoft, Redmond, WA, USA). Statistical significance was determined to be p < 0.05.

## Results

### Changes in the morphology of SH-SY5Y cells after application of RA and RA + NTP

**Figure 2:**
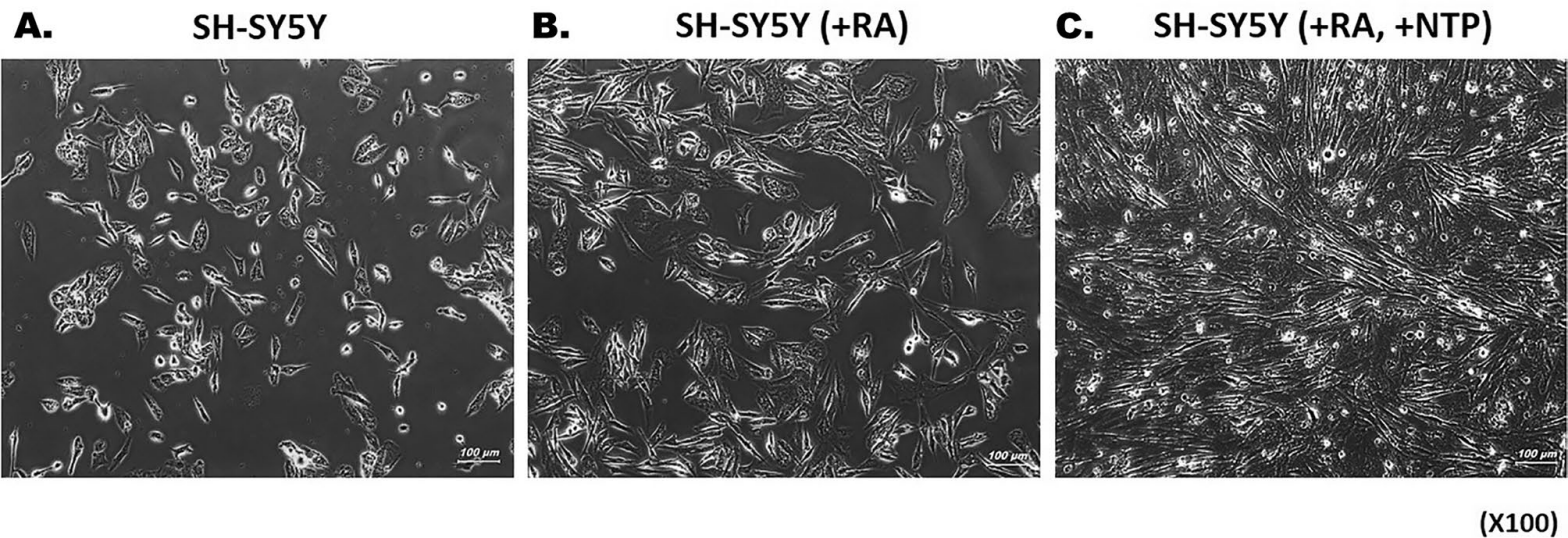
Optical microscope images of SH-SY5Y cells before and after RA/NTP treatment. (**A**) Cells before RA application (Undifferentiated normal SH-SY5Y Cell). (**B**) After 7 days of RA application, the extension of neurites was seen (Cells are differentiated). (**C**) After the NTP treatment on SH-SY5Y cells, the number of cells increased, and axonal elongation progressed further (NTP was processed 3 times a week).

Images of the SH-SY5Y cells and the cells after application of RA and RA/NTP were obtained using an optical microscope. Compared with their appearance before RA application (Fig. 2A), cells exhibited neurites extension, a typical neuronal phenotype, following the application of RA (Fig. 2B). After NTP treatment, the number of cells increased, and axonal elongation progressed (Fig. 2C).

### Expression of tau protein level by NTP in differentiated SH-SY5Y cells via Western Blot

**Figure 3:**
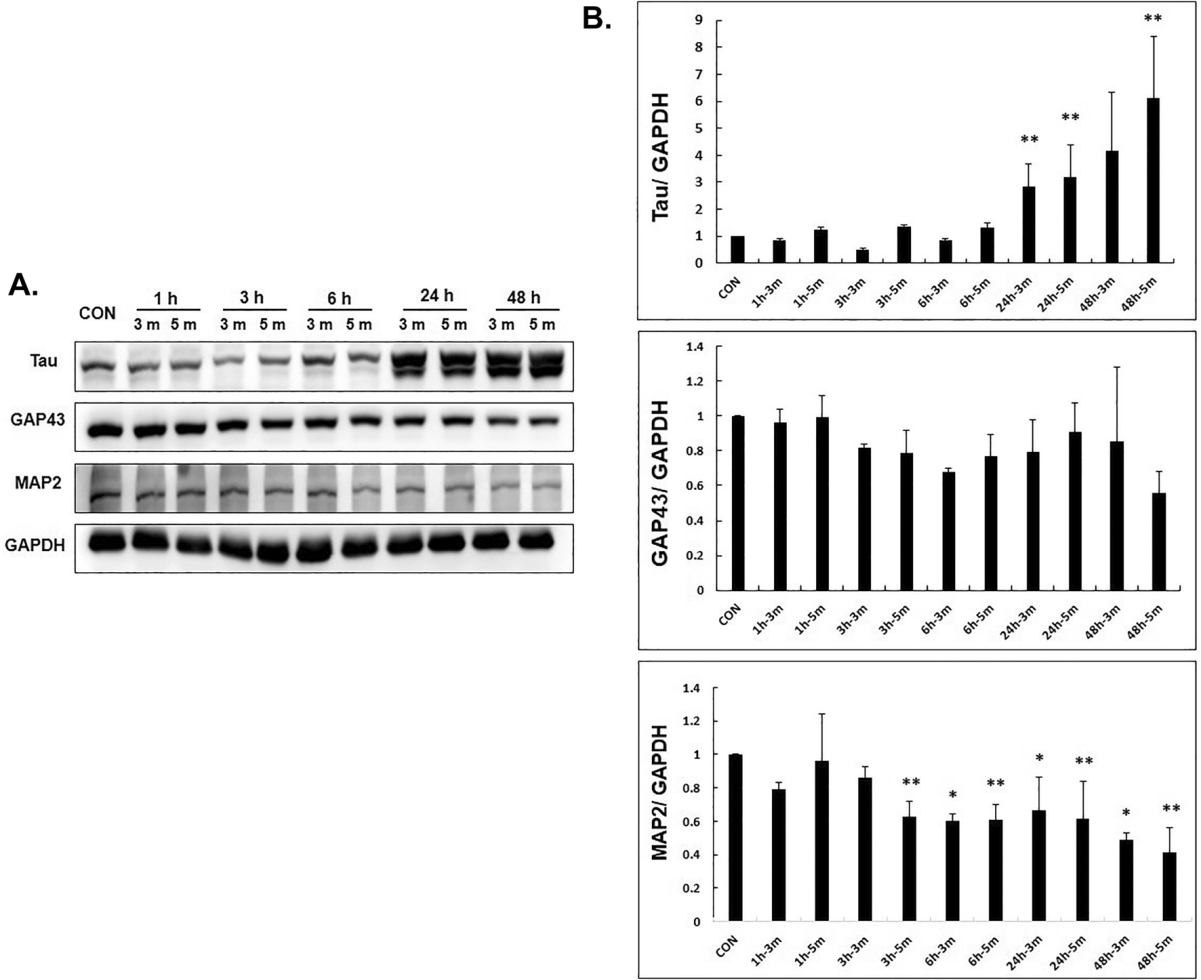
Western blotting of SH-SY5Y cell extracts. GAPDH was used as a loading control. The expression of tau increased significantly after 24 h of NTP treatment. The expression of GAP43 showed no significant difference, while the expression of MAP2 decreased significantly after 3, 6, 24, and 48 h of NTP treatment. *Indicates *p* < 0.05, ** indicates *p* < 0.01.

When SH-SY5Y cells were treated with NTP at different time intervals, the effect on the factors related to nerve regeneration was evaluated (Fig. 3).

After treatment with NTP in SH-SY5Y cells for 3 min and 5 min respectively, samples were obtained after 1, 3, 6, 24, and 48 h, and western blot was performed. (Fig. 3A).

It was seen that the expression of tau increased at 24 h and 48 h after NTP treatment. There were no significant difference in the expression of GAP43 whereas the expression of MAP2 gradually decreased over time after NTP treatment.

Each band was quantified with GAPDH and the significance was confirmed by drawing a graph (Fig. 3B). The expression of tau was significantly increased at 24 and 48 h after NTP treatment.

It was confirmed that the expression of MAP2 was significantly reduced at 3, 6, 24 and 48 h after NTP treatment.

### Changes in wnt signaling at mRNA level by NTP in differentiated SH-SY5Y cells via RT-PCR

**Figure 4:**
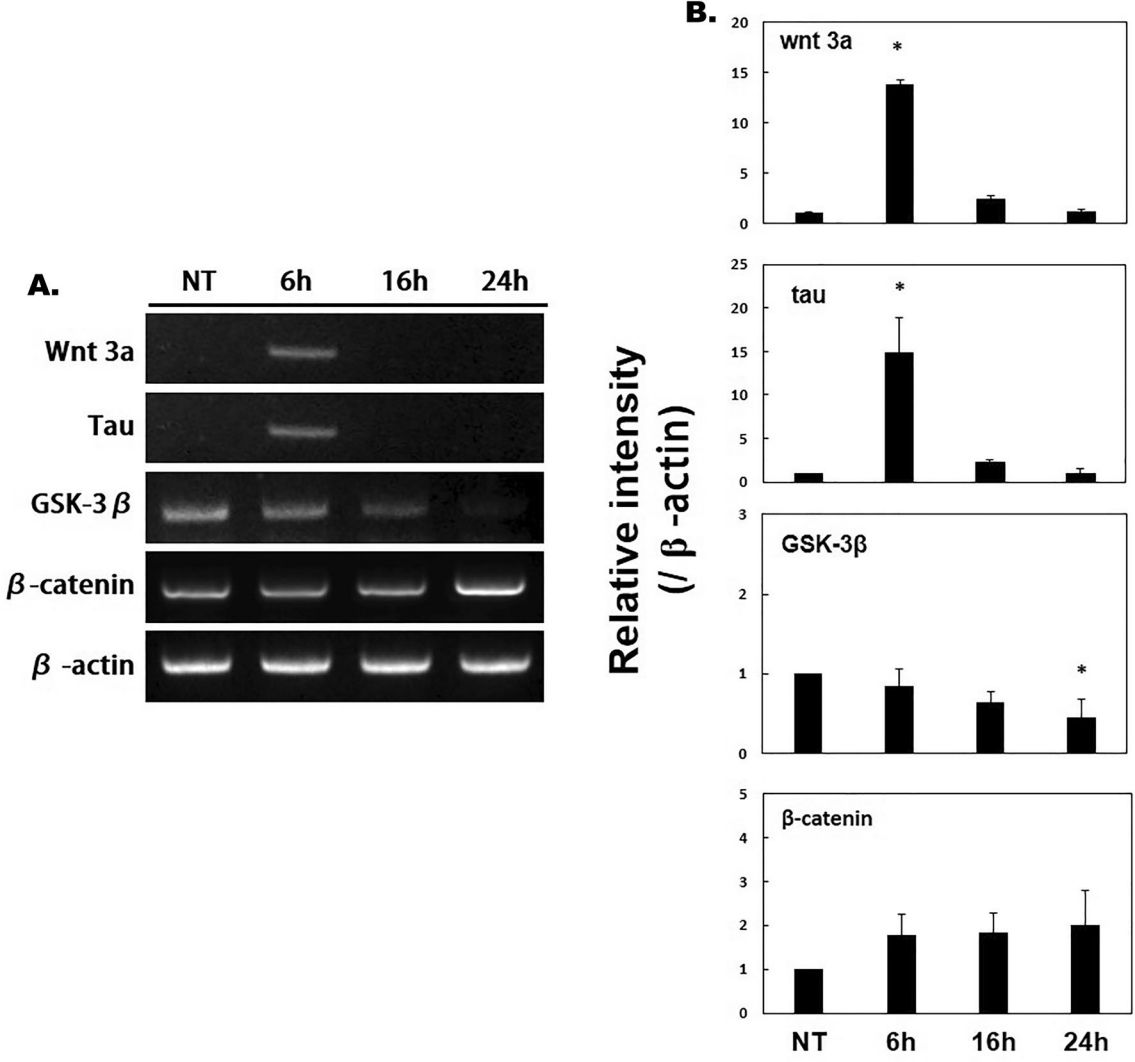
RT-PCR of SH-SY5Y cell extracts. Tau and wnt3a were highly expressed after 6 h of NTP treatment. β-catenin was highly expressed after 24 h of NTP treatment. GSK3β expression was reduced after 24 h of NTP treatment. *Indicates *p* < 0.05.

After NTP treatment on SH-SY5Y cells, RT-PCR was performed after 6, 16, and 24 h (Fig. 4A).

The gene expression of Tau and wnt3a was confirmed 6 h after NTP treatment. GSK3β showed a pattern of gradually decreasing gene expression after NTP treatment. And the gene expression of β-catenin seemed to increase gradually over time, and in particular, the gene expression increased the most after 24 h of NTP treatment (Fig. 4A).

The bands of RT-PCR were quantified with β-actin and presented as a graph (Fig. 4B). It was confirmed that the gene expression of wnt3a, tau was significantly increased when 6 h passed after NTP treatment. It was confirmed that the expression of GSK3β was significantly reduced after 24 h of NTP treatment.

### Confirmation of localization of tau by NTP via fluorescence staining

**Figure 5:**
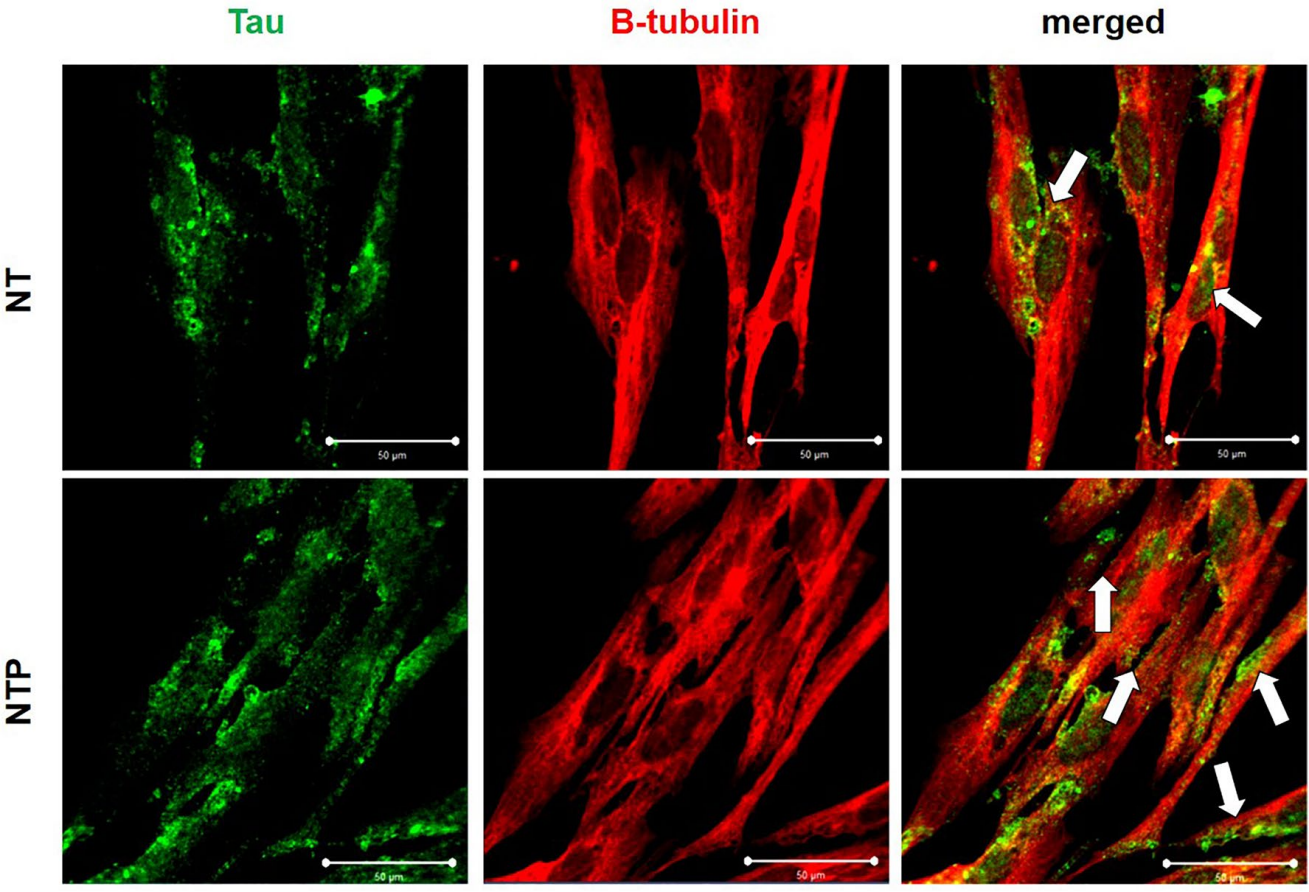
Immunofluorescence assay images. Tau and β-tubulin expression were detected with a confocal microscope. Tau was observed to be located around the nucleus in the NT group, while the expression of tau was increased around the axons in the NTP group (white arrow).* Scale bars = 50 μm.

Merged images of green-stained tau and red-stained β-tubulin were acquired (Fig. 5). In the NT group, tau was observed to be located around the nucleus (Fig. 5C). However, in the NTP group, it was confirmed that the amount of tau was increased as it extended to the axons (Fig. 5F).

### Confirmation of changes in the expression of factors related to nerve regeneration by NTP through animal experiments

**Figure 6:**
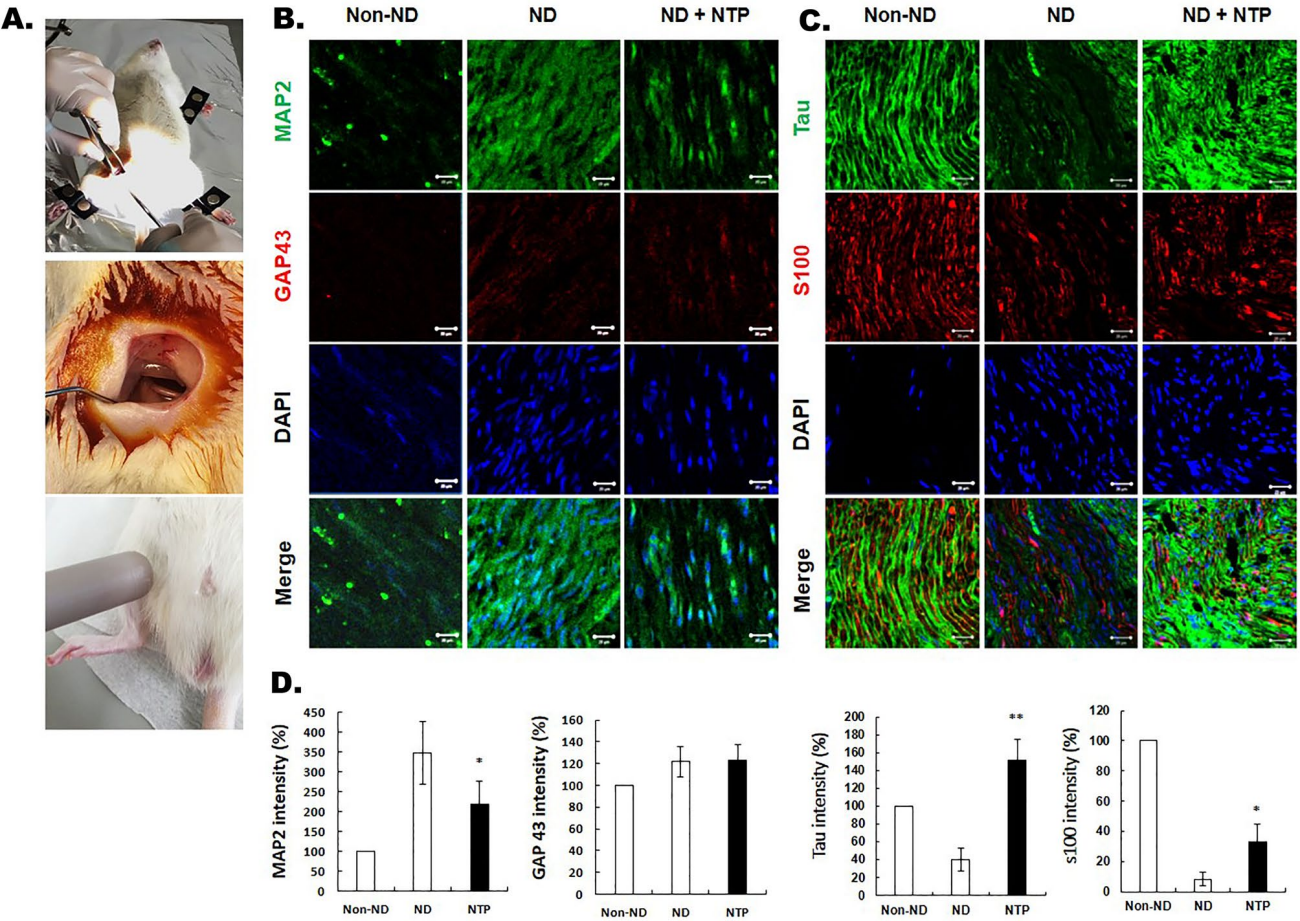
Animal experiments and results. (**A**) Image of surgery for anastomosis of cut sciatic nerve and NTP treatment of the damaged nerve in the rat model. NTP was applied 0.5 cm away from the skin overlying the sciatic nerve for 5 min. (**B**) and (**C**) Immunofluorescence of neural tissue in the three groups and quantitative analysis: MAP2, GAP43, tau, S100B, and DAPI. Representative photographs of each group were taken using confocal microscopy. *Scale bars = 20 μm. *Indicates *p* ≤ 0.05, ** indicates *p* ≤ 0.01.

As seen in the immunofluorescence analysis, MAP2 was overexpressed in the ND group compared to the non-ND group. However, in the ND + NTP group, the expression of MAP2 was decreased significantly than in ND group. GAP43 expression was not significantly different in all three groups (Fig. 6B).

The expression of tau was decreased in ND group than non-ND group. However, in ND + NTP group, the expression of tau was increased significantly than ND group. S100B was less expressed in the ND group than in other groups. However, the expression of S100B in the ND + NTP group was increased significantly than in the ND group (Fig. 6C).

## Discussion

In this study, SH-SY5Y cells were used to investigate the effect of NTP on neurons. SK-N-SH cell, the original cell line, was subcloned and isolated from a bone marrow biopsy acquired from a 4-year-old girl with a neuroblastoma. As documented in numerous studies, fully differentiated neuron-like cells can be obtained from SH-SY5Y neuroblastoma cell lines by treatment with RA^17,18^. SH-SY5Y cells must be differentiated for minimum of 7 days for experimentation^19^. Neurons then need to be kept in culture for minimum of 1 week to show mature excitability and action potential propagation. In this study, RA was treated for 7 days to induce differentiation and subsequently, SH-SY5Y cells were differentiated into neuron-like cells.

In the images obtained by optical microscopy (Fig. 2), neuron-like cells exposed to NTP showed a higher cell count and more active axonal formation. Images showed that NTP accelerated neuronal cell proliferation.

Western blot analysis was performed to determine whether NTP treatment of cells increased the level of protein that induces neuronal proliferation. Expression of tau was increased significantly after 24 h of NTP treatment. tau is mainly distributed in the axons of neurons, and its primary function is maintaining the stability of microtubules in axons^20^. Several previous in vitro studies have confirmed that tau promotes microtubule nucleation, growth, and bundling^8,9^ and can dramatically reduce the dynamic instability of microtubules^21^. In particular, tau can influence net microtubule assembly, the rate of neurite elongation, and neuritic stability^22^. This indicates that tau plays an important role in the development and maintenance of neurons.

The expression of GAP43 was not significantly different between NTP treatment and control. GAP43 is present in high levels and is associated with the surface plasma membranes of developing and regenerating axons, especially those of growth cones^23^. Aigner et al.^24^ reported that aberrant neuronal connections develop in transgenic mice overexpressing GAP43. On the other hand, Strittmatter et al.^25^ reported that complete GAP43 knockdown in mice is fetal in 90% of the all cases during the first 3 weeks of postnatal life, which suggests that GAP43 is crucial for neuronal network formation. In this study, the level of GAP43 was maintained, which indicated that aberrant axonal sprouting due to overexpression of GAP43 was suppressed.

MAP2 had decreased significantly following NTP treatment. MAP2 is thought to be associated with an increase in dendrite during neuronal regeneration. Moreover, the overexpression of MAP2 may be implicated in the formation of neuromas^26–28^. This suggests that NTP could prevent the formation of neuroma following injury.

To identify the factor that induces neuronal proliferation at the mRNA level, RT-PCR was performed, and it was confirmed that the expression of tau had also increased, which was consistent with the results of the western blot. Expression of wnt3a had increased significantly after 6 h and β-catenin had increased significantly after 24 h of NTP treatment. On the contrary, expression of GSK3β had decreased significantly after 24 h. Increased levels of wnt3a and β-catenin indicate their involvement in cell proliferation. β-catenin moves into the nucleus and regulates cell proliferation by regulating the expression of various genes. In the absence of stimulation by Wnt, β-catenin is phosphorylated and decomposed by casein kinase I and GSK3β, and the cytoplasmic β-catenin levels are kept low. However, when Wnt stimulation occurs from outside the cell, the activity of GSK3β is inhibited, and the phosphorylation of β-catenin is also inhibited. As cytoplasmic β-catenin levels increase and move into the nucleus, it binds to the transcription factor, Tcf, and regulates the expression of several genes. This study suggested that increased wnt3a induces an increase in β-catenin and that increased β-catenin promotes cell proliferation.

Immunofluorescence analysis was used for determining the intracellular location of tau. In this experiment, tau was shown to be deposited along the axon during immunofluorescence following NTP treatment. This indicated that increased level of tau after NTP treatment promoted axonal elongation and microtubule stabilization.

The immunofluorescence assay in vivo showed that MAP2 was overexpressed in ND group, whereas in ND + NTP group, expression was similar to that of non ND grop. Futhermore, GAP43 did not show any significant difference between the ND + NTP and non-ND groups. These results are consistent with those of the in vitro experiments, which reconfirm that NTP inhibits the side effects of neuronal regeneration that occur when MAP2 and GAP43 are overexpressed. Additionally, as observed in the in vitro experiments, NTP led to an increase in tau and S100B, which affect axonal elongation.

The results of this study suggest that there are several mechanisms for the acceleration of neuronal proliferation by NTP. First, NTP accelerates neuronal proliferation by increasing the expression of tau. tau plays an important role in the development and maintenance of neurons. Evidence for the participation of tau in axonal growth has been studied. Brandt et al., demonstrated that tau is capable of promoting microtubule nucleation, growth, and bundling^29,30^, and also of dramatically reducing the dynamic instability of microtubules^21^. Esmaeli-Azad et al.^22^ investigated the role of tau in several aspects of neuronal behavior using PC12, a nerve growth factor-responsive cell line. They found that tau-suppressed PC12 cells display slower neurite outgrowth, reduced microtubule levels, and lesser resistance to nocodazole-induced neurite retraction. However, stable transfectants overexpressing tau accumulate more microtubule mass and extend neurites more rapidly than control cells. The results from the current study showed an increase in tau following NTP treatment, which is presumed to have enhanced neurite outgrowth and stabilization of microtubules.

Futhermore, NTP inhibits overexpression of MAP2 and GAP43, thereby preventing possible side effects during neuronal proliferation. Overexpression of MAP2 may be associated with neuroma formation, and overexpression of GAP43 may lead to aberrant axonal sprouting. NTP treatment contributes to neuronal proliferation by preventing overexpression of these factors.

Another possible mechanism is that neuronal proliferation is promoted through increased Wnt. Wnt is a family of signaling proteins involved in various developmental functions, ranging from morphogenesis to, cell proliferation, and survival^31^. Wnt binds to various receptors and activates other downstream pathways. These pathways have been classified as canonical (β-catenin dependent) or non-canonical (β-catenin independent) signaling pathways. Via the canonical pathway, Wnts induces activation and nuclear translocation of β-catenin through inhibition of GSK3β^32–34^, and β-catenin moves into the nucleus and regulates cell proliferation by regulating the expression of various genes. Moreover, wnt1 and wnt3a have functions that promote cell proliferation in the neural tube^32,33^. In a previous study, the absence of wnt1 and wnt3a, which are normally expressed in the roof plate, was shown to diminish the development of dorsal interneurons D1 and D2 and increase D3 populations in compensation^34^. Wnt3a also promotes neuronal regeneration following traumatic brain injury. Chang et al. demonstrated that recombinant wnt3a protein promotes neuronal regeneration and functional recovery^35^. Regeneration of damaged cortical neurons has been shown to improve by 20–25% with wnt3a as compared to regeneration without wnt3a. In the current study, wnt3a and β-catenin significantly increased after NTP treatment, which confirmed that nerve regeneration was facilitated through Wnt signaling.

This study, in which SH-SY5Y cells differentiated into neuron-like cells were treated with NTP, showed that:

1. The number of nerve cells increased, and axonal elongation progressed. Western blot, RT-PCR, and immunofluorescence assays showed an increase in tau, which can accelerate neuronal proliferation.
2. RT-PCR assays indicated that the expression of wnt3a and β-catenin was significantly raised. Increased levels of wnt3a altered β-catenin activity through Wnt signaling. Thereby, upregulated β-catenin promoted cell proliferation.
3. The expressions of MAP2 and GAP43 were not different or reduced before and after NTP treatment according to western blot and immunofluorescence assays, respectively, in vivo. NTP inhibited the overexpression of MAP2 and GAP43, which can negatively affect neuronal proliferation.

The present study showed that the treatment of NTP accelerated neuronal proliferation of human neuron-like cells. This would provide the basis for studying NTP as a noninvasive and effective treatment method for nerve injury caused by trauma or disease.

## Supplementary Information

Supplementary Information 1.

Supplementary Information 2.

Supplementary Information 3.

Supplementary Information 4.

## Data Availability

All data generated or analyzed during this study are included in this article (and its Supplementary Information files).
