# Supplementary material for: Non-thermal plasma directly accelerates neuronal proliferation by stimulating axon formation
Source: Sci Rep. 2022 Sep 23;12:15868. doi: 10.1038/s41598-022-20063-4 (PMC9508269; doi:10.1038/s41598-022-20063-4)

The original file of figure 3

(In addition to the protein size marker, the molecular weight of each antibody and the location of expression in the blot are indicated.)

1. GAP43- 43 kDa

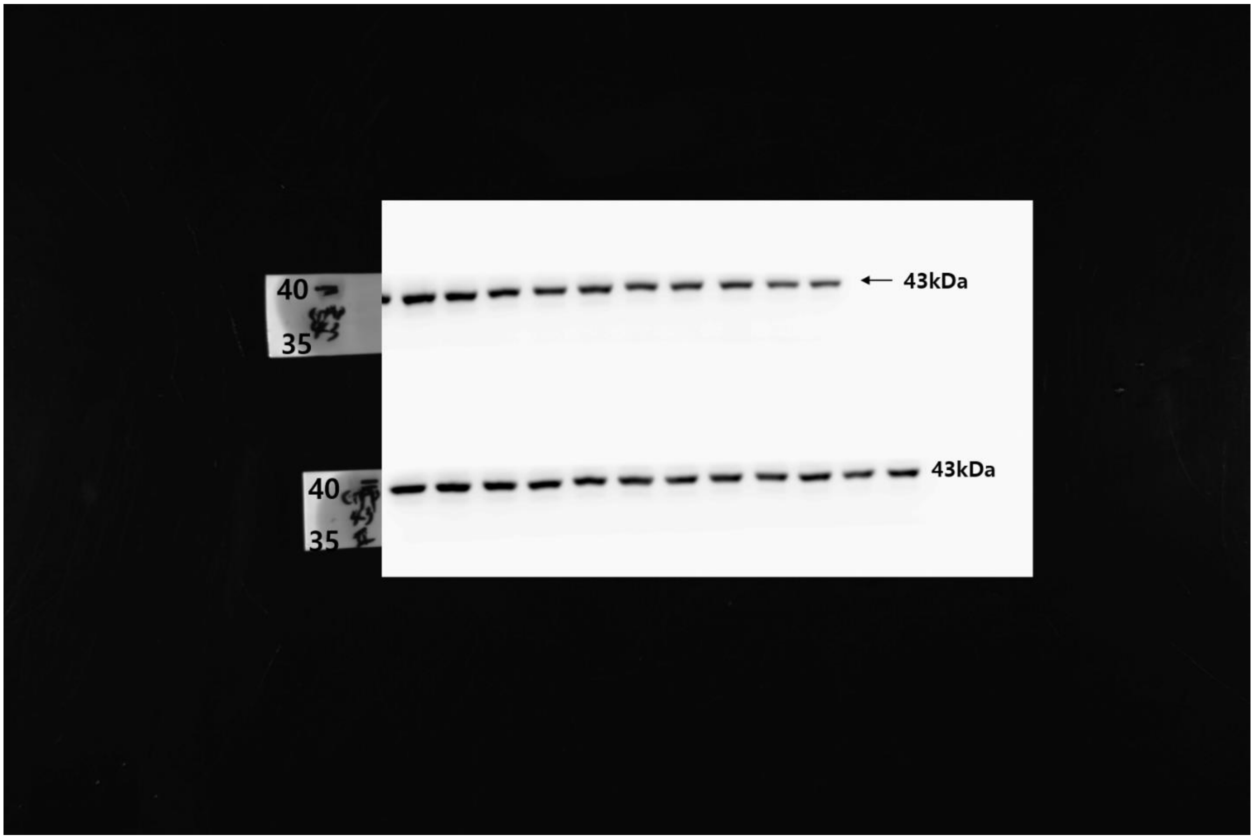

2. MAP2- 280 kDa

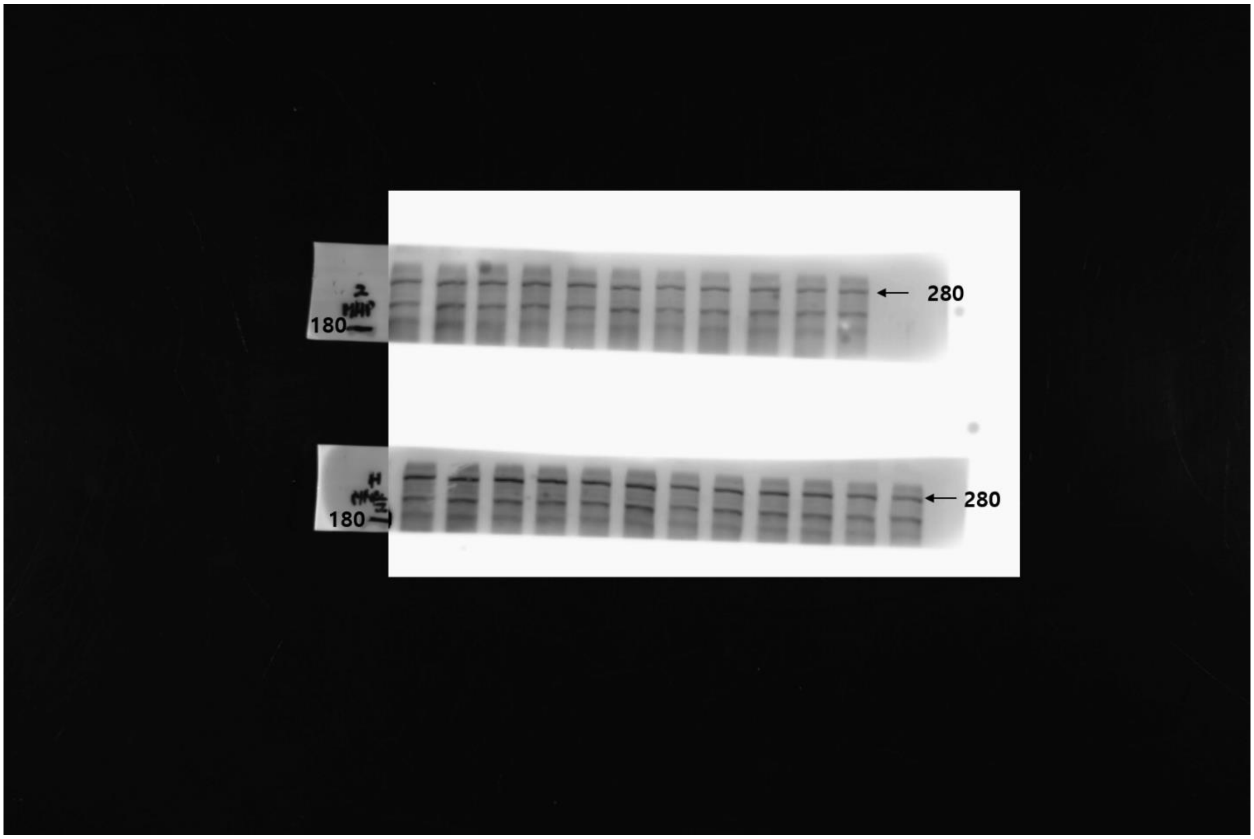

3. tau- 79 kDa

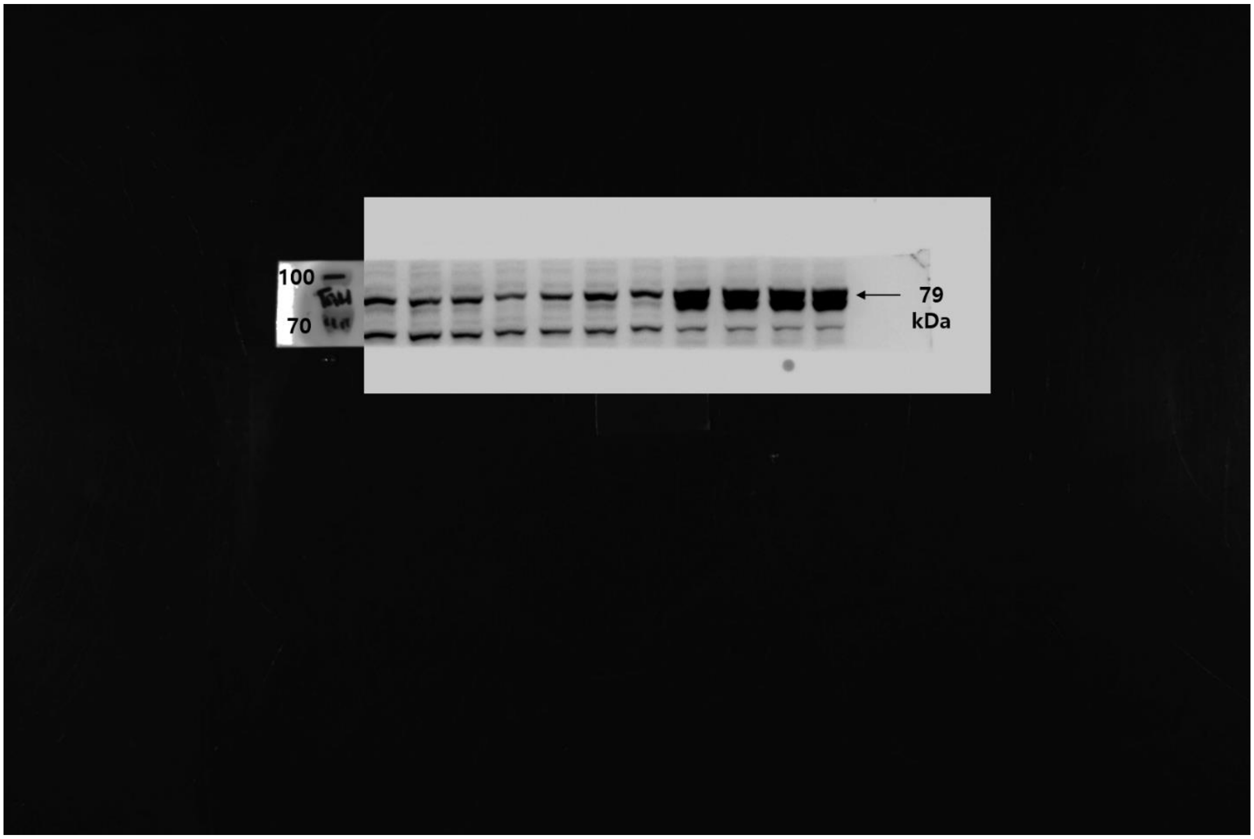

4. GAPDH- 36 kDa

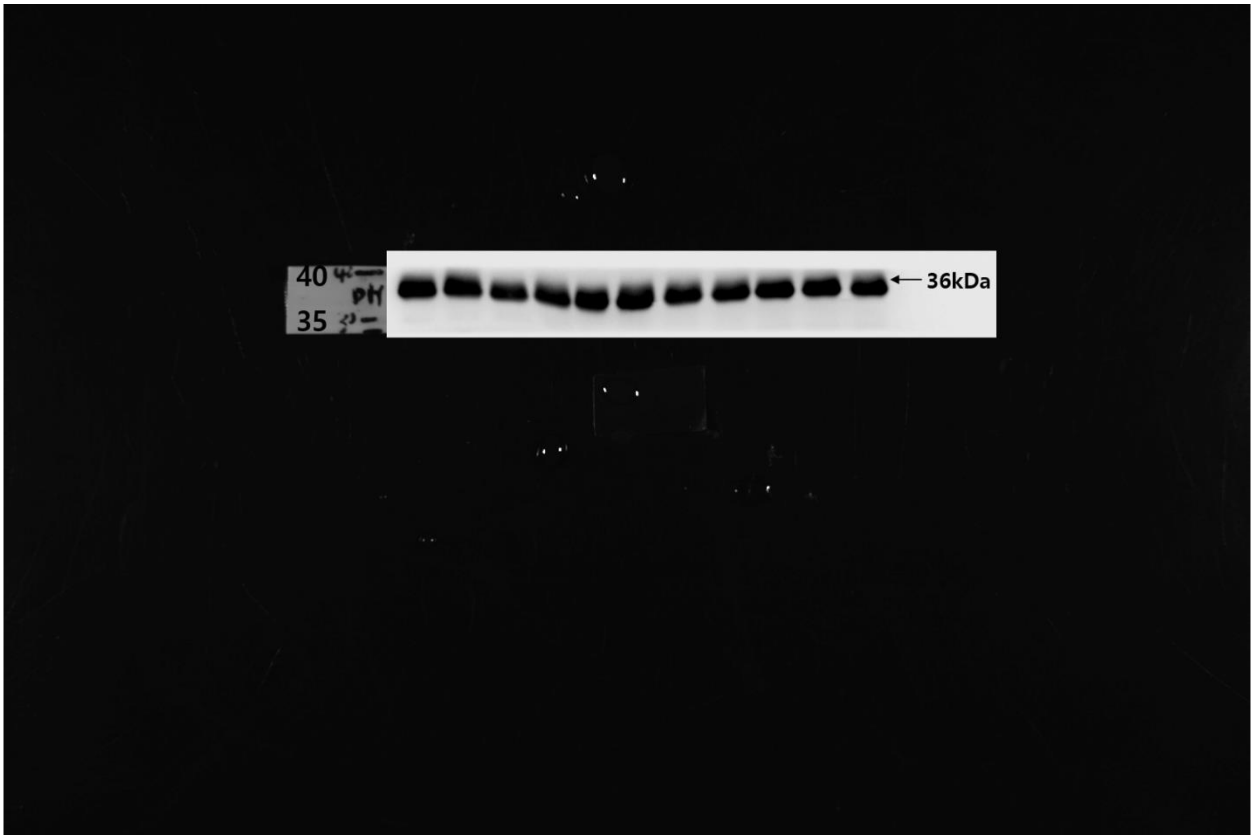

Supplement: Supplementary file 1 — Supplementary Information 1. [file 41598_2022_20063_MOESM1_ESM.pdf]
