# Supplementary material for: Non-thermal plasma directly accelerates neuronal proliferation by stimulating axon formation
Source: Sci Rep. 2022 Sep 23;12:15868. doi: 10.1038/s41598-022-20063-4 (PMC9508269; doi:10.1038/s41598-022-20063-4)

While performing RT-PCR, several primers were added to the gel and the results were confirmed. The reason behind this was to save the cost and time of the experiment. As we know that, RT-PCR is an expensive process. So, we added several primers to the different gels according to the suitability.

Hereby, RT-PCR original images are added.
In the original gel of RT-PCR, other primers are mixed in addition to each primer. Therefore, according to the position of the primer confirmed in the Figure 4, we have marked (Red square) the specific primers that we used in the respective original images.

1. b-actin

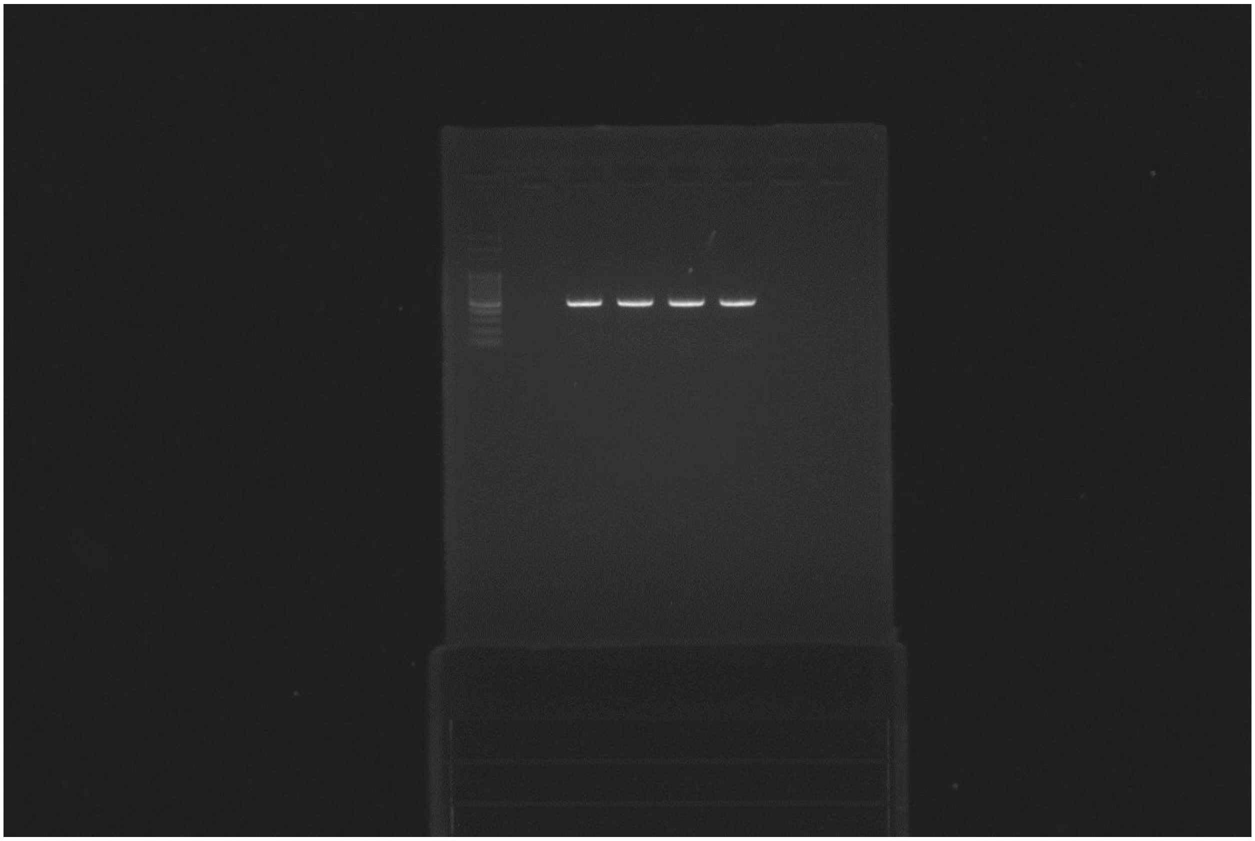

2. beta-catenin (right band, #10-13)- original image

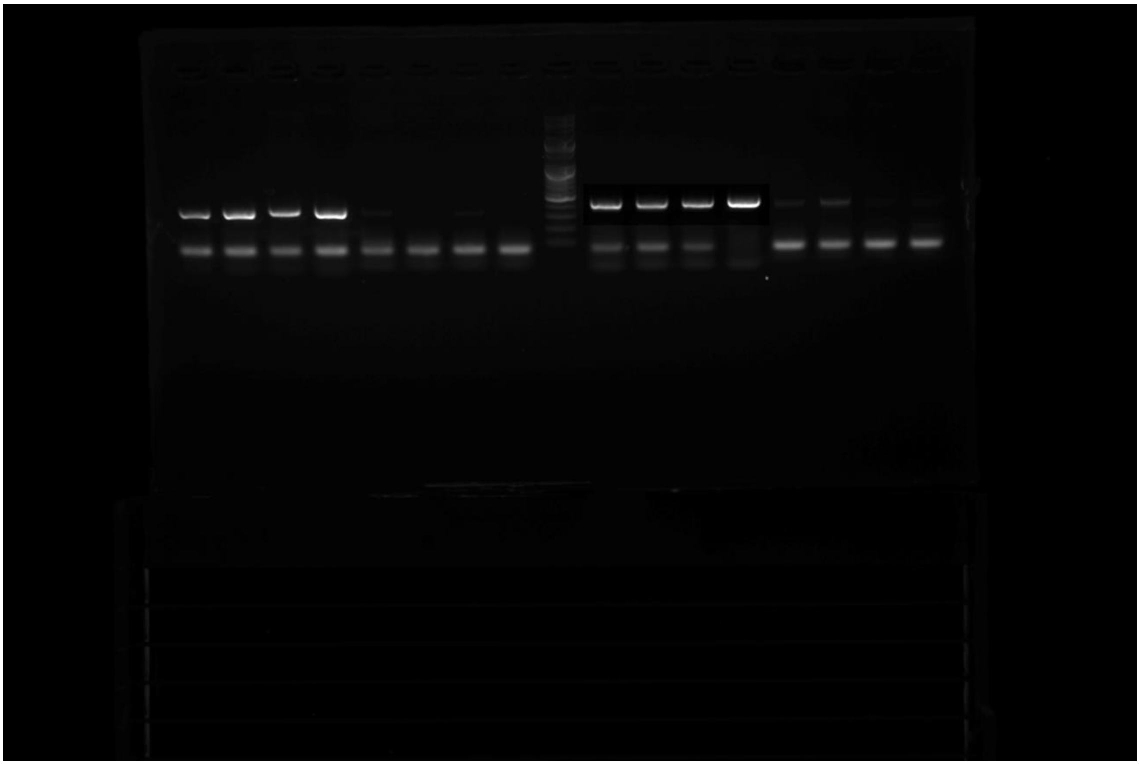

beta-catenin - Red square: the position of the primer we used

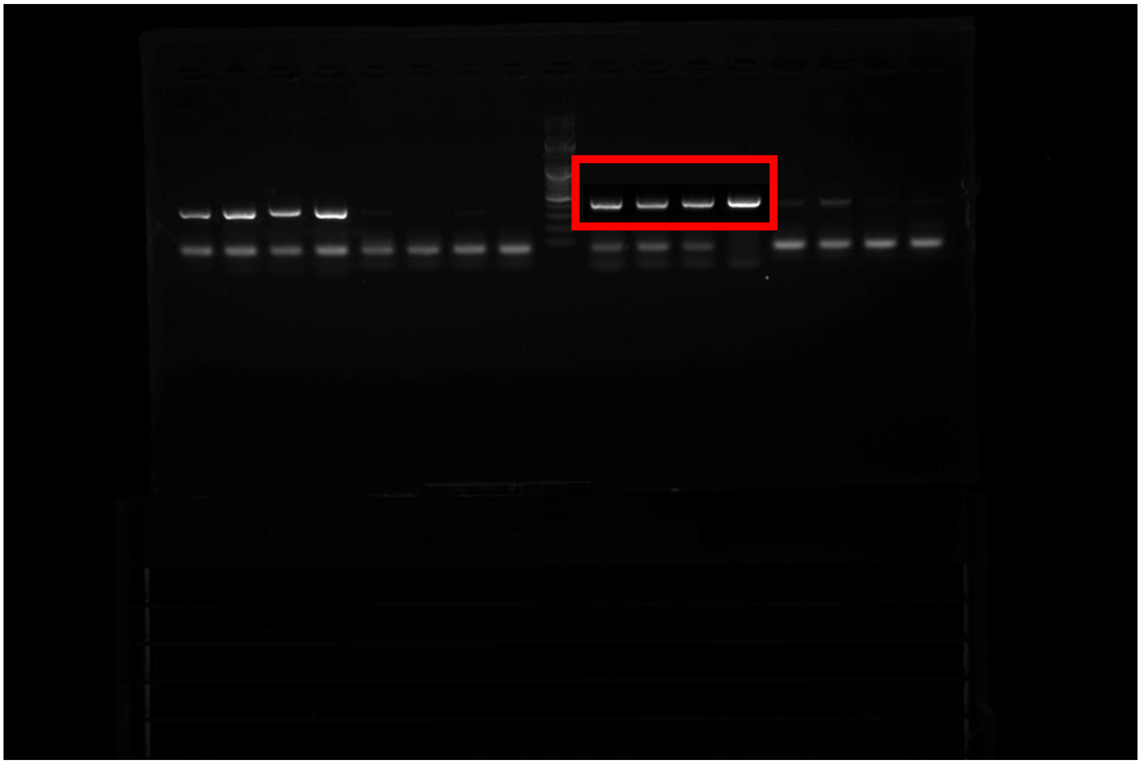

3. GSK-3 beta (left band, #1-4)- original image

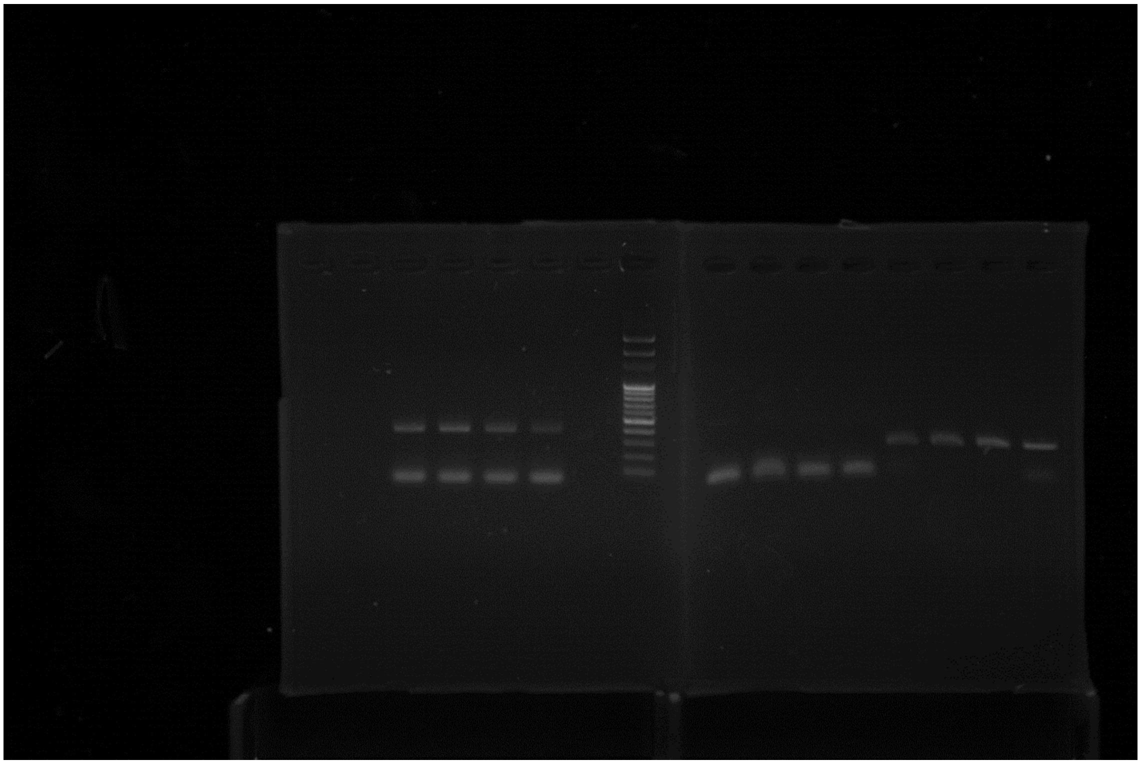

GSK-3 beta (left band, #1-4) - Red square: the position of the primer we used

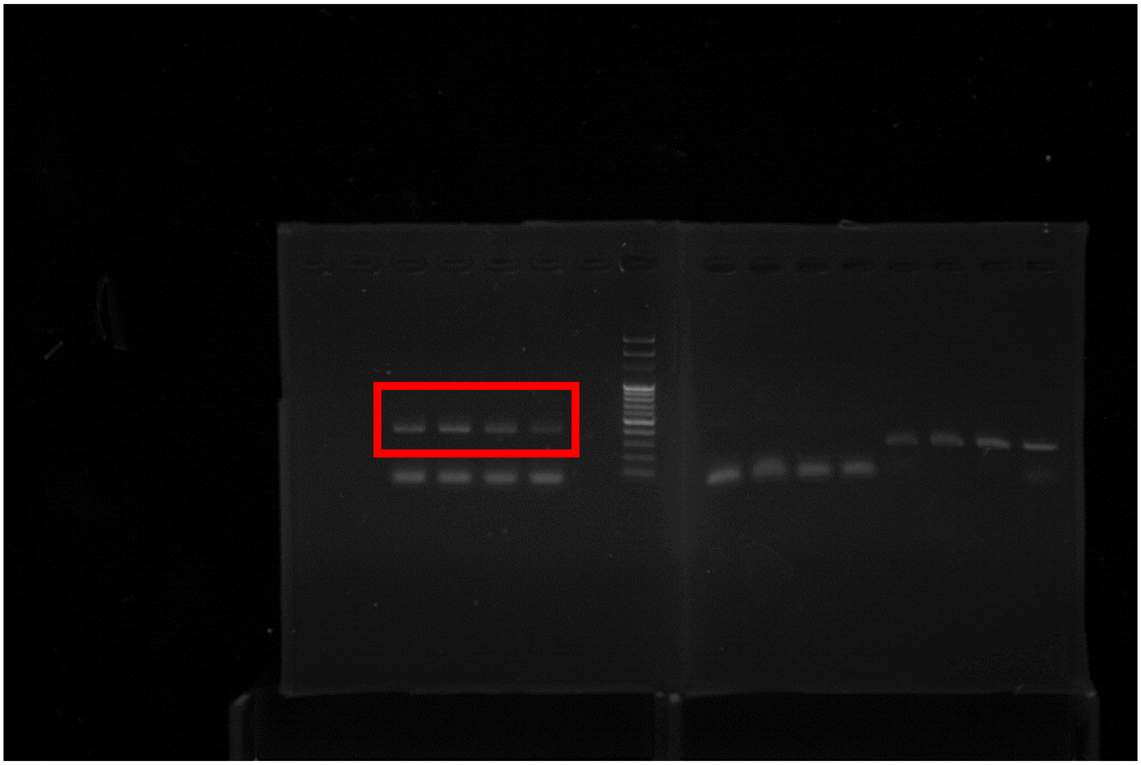

4. TAU (6th band)) - original image

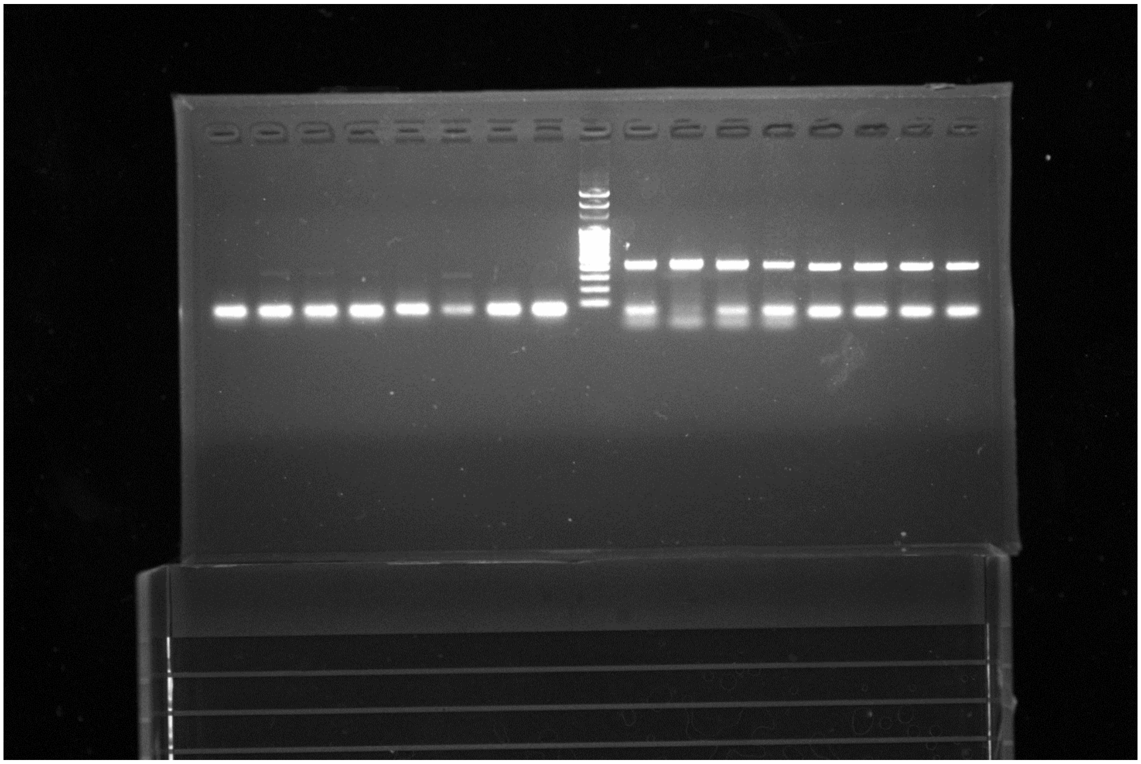

TAU (6th band) - Red square: the position of the primer we used

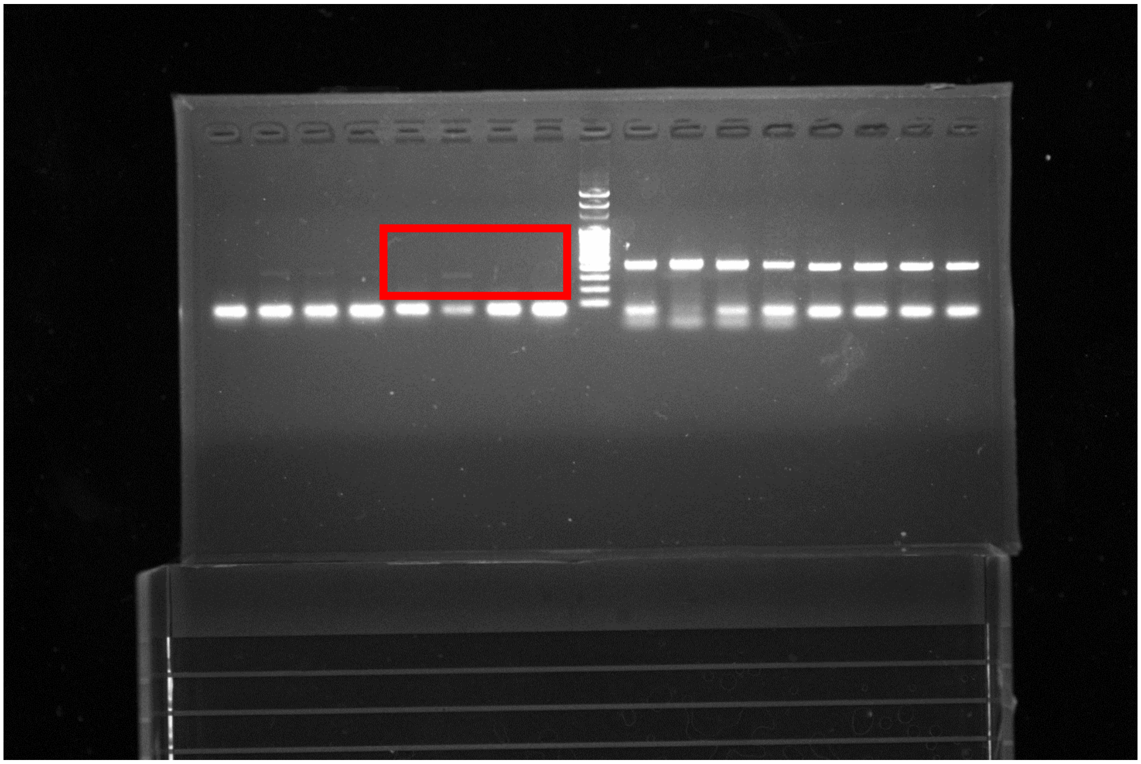

5. Wnt3a (right band, #15)- original image

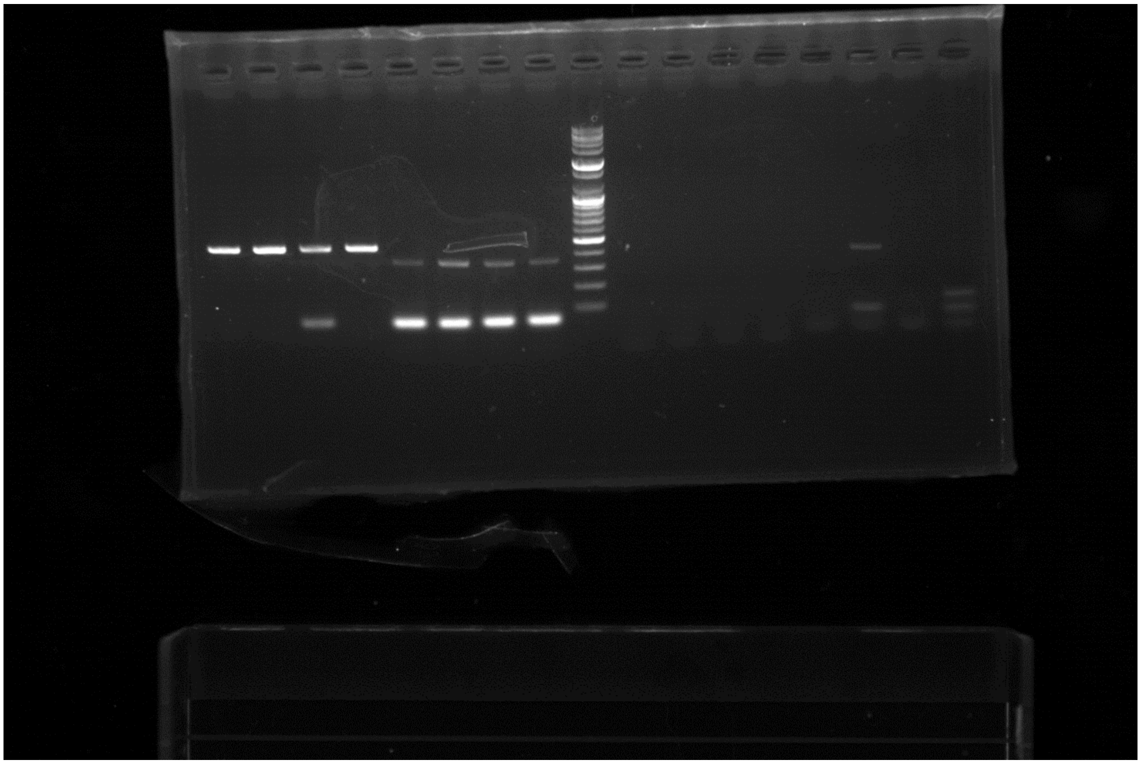

6. Wnt3a (right band, #15) - Red square: the position of the primer we used

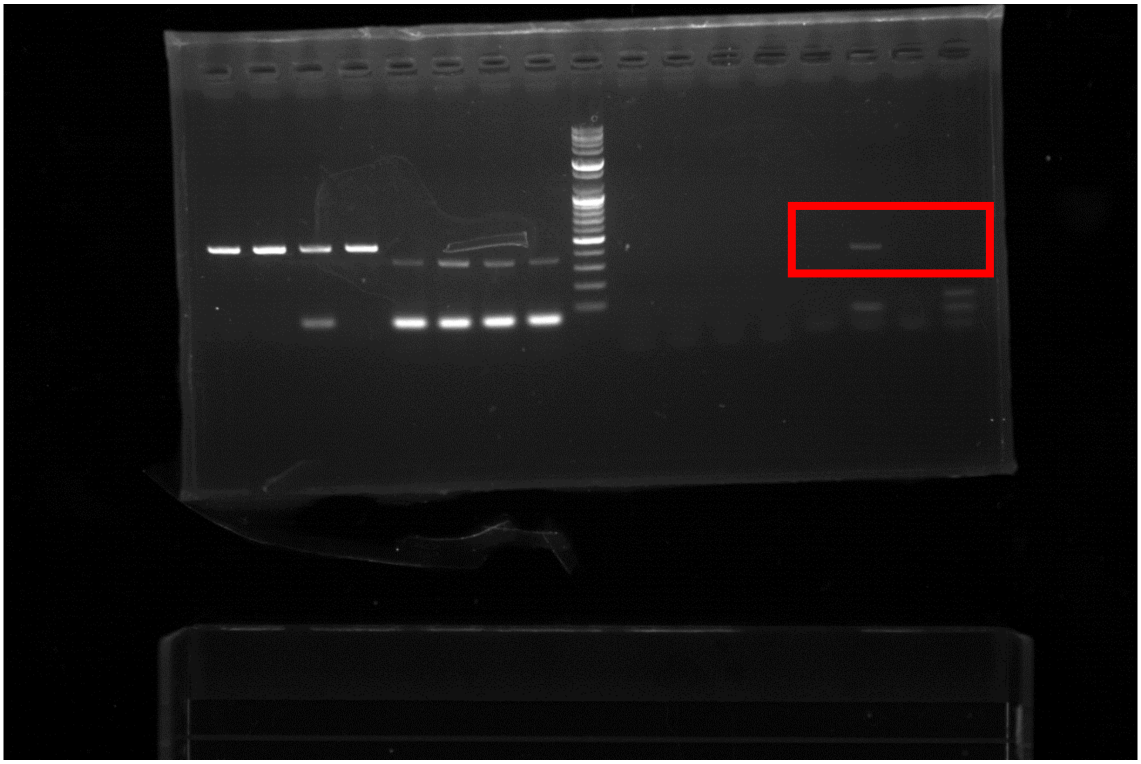

Supplement: Supplementary file 2 — Supplementary Information 2. [file 41598_2022_20063_MOESM2_ESM.pdf]
