# Supplementary material for: Non-thermal plasma directly accelerates neuronal proliferation by stimulating axon formation
Source: Sci Rep. 2022 Sep 23;12:15868. doi: 10.1038/s41598-022-20063-4 (PMC9508269; doi:10.1038/s41598-022-20063-4)
Supplement: Supplementary file 3 — Supplementary Information 3. [file 41598_2022_20063_MOESM3_ESM.pdf]

Figure 2. Cells Images

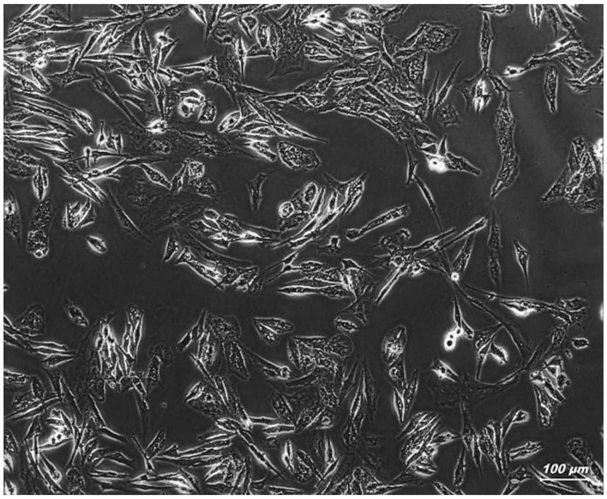

SH-SY5Y(+RA)

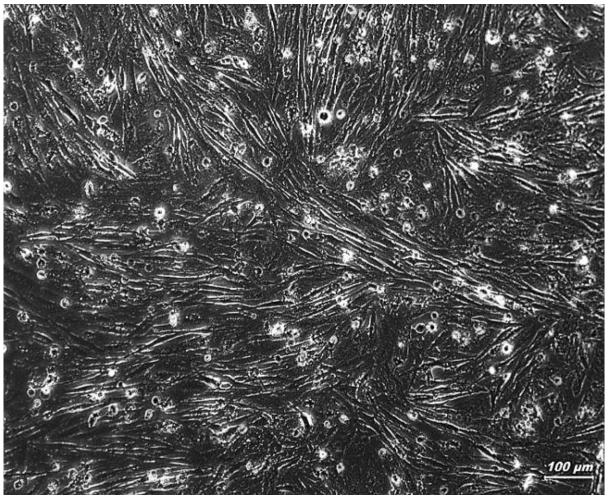

SH-SY5Y(+RA+NTP)

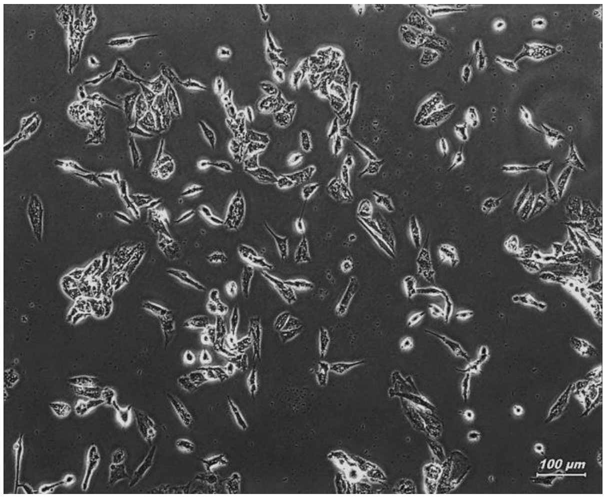

SH-SY5Y

Figure 4. RT-PCR

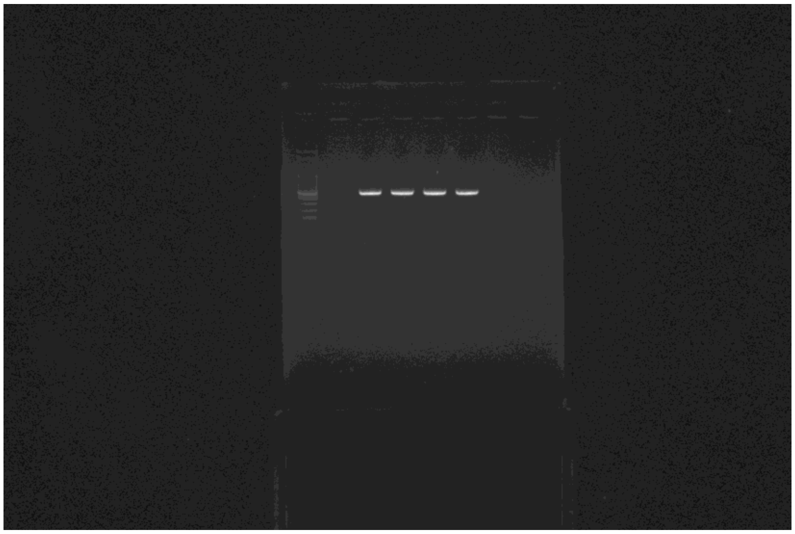

ACTIN

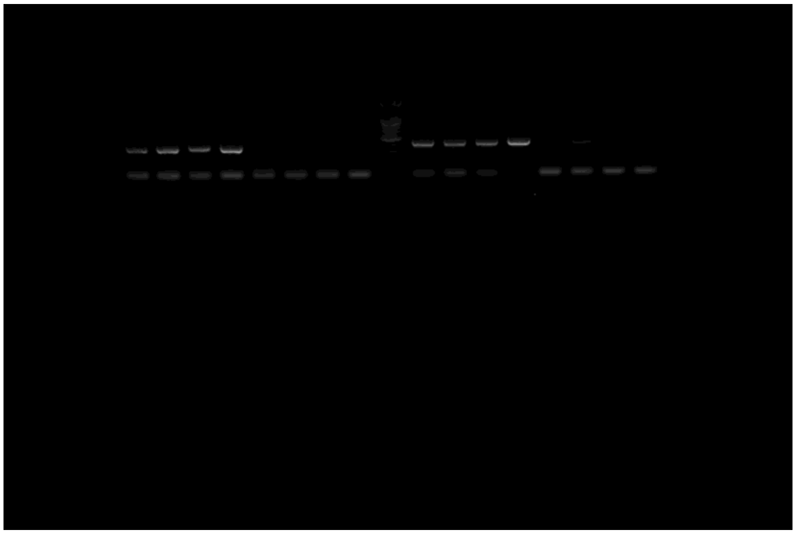

B-CATENIN

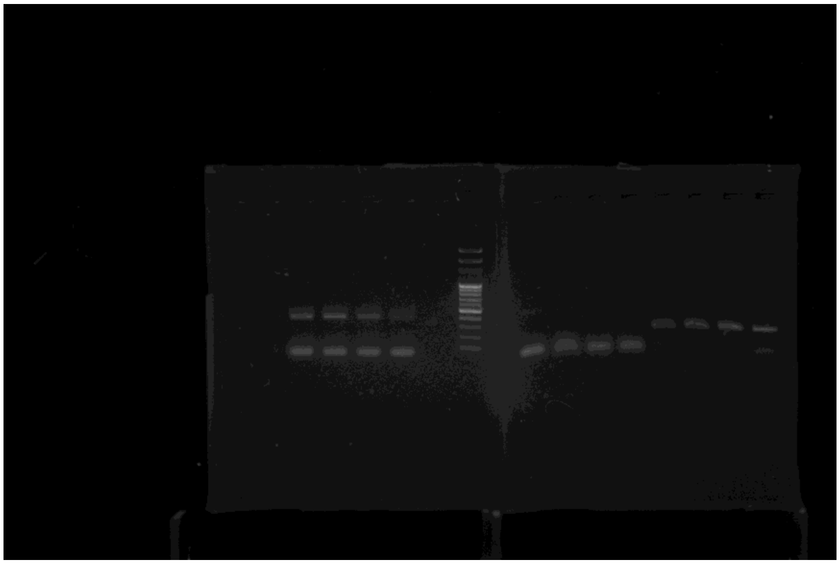

GSK-3B

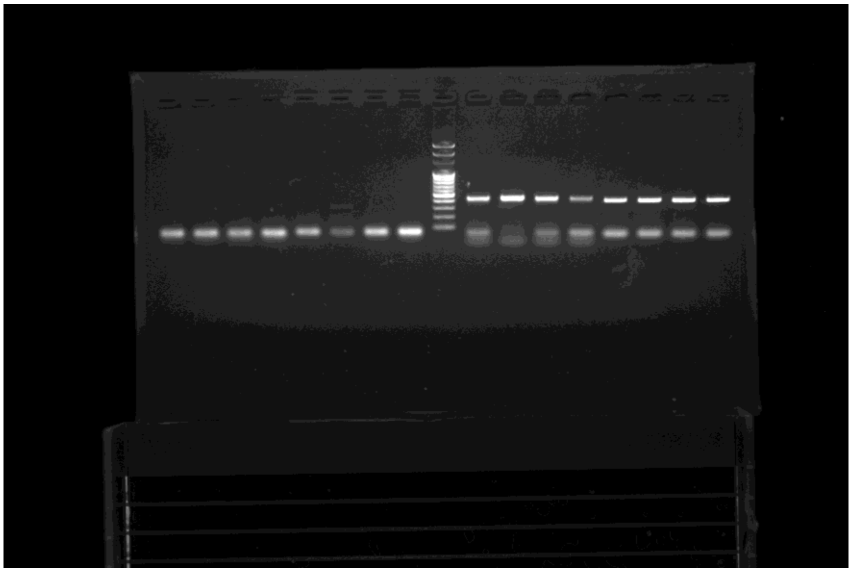

TAU

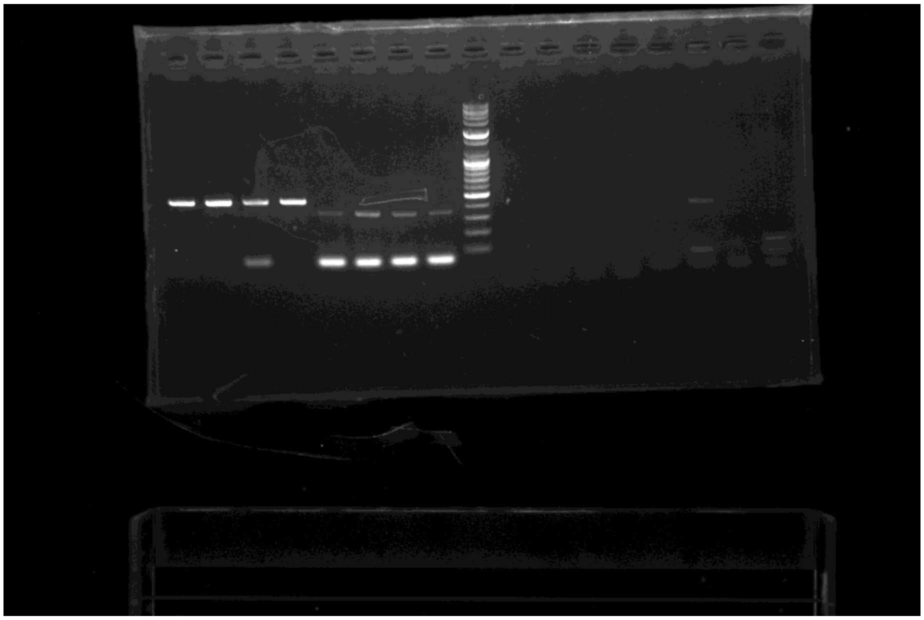

WNT3

Figure 5. TAU, b-TUBULIN IF

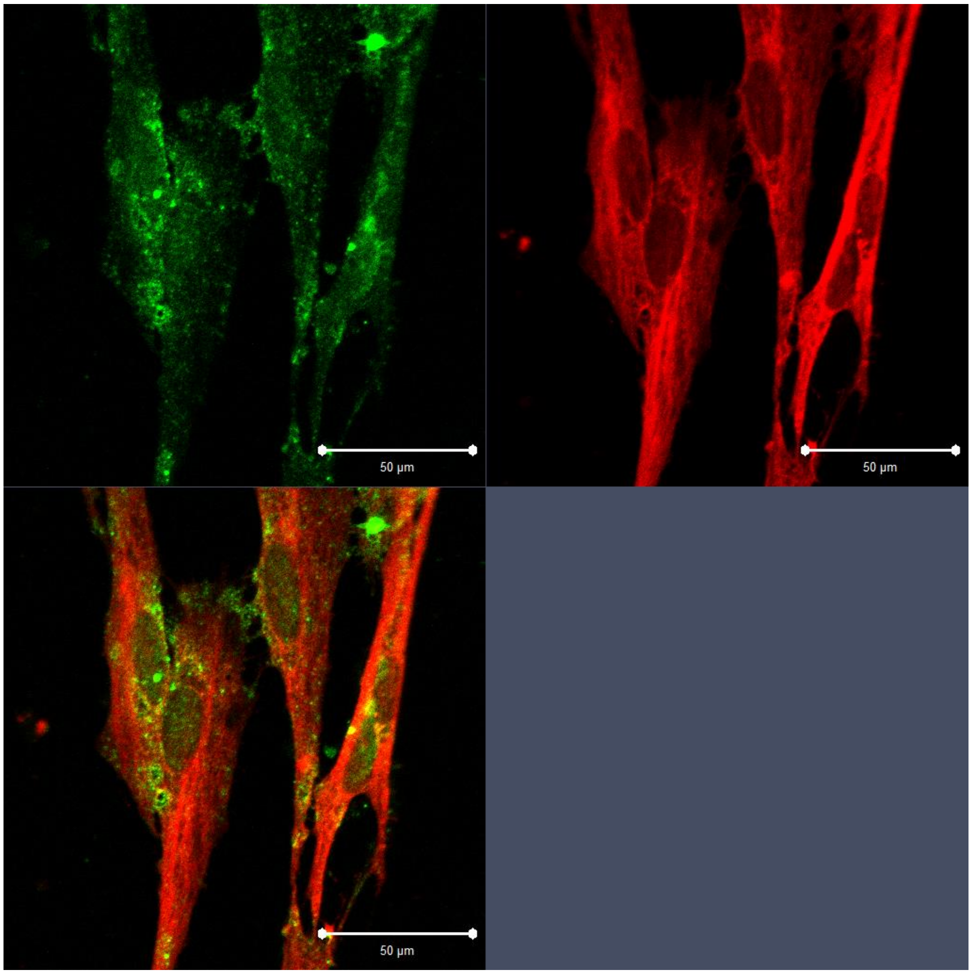

X40-NT-2

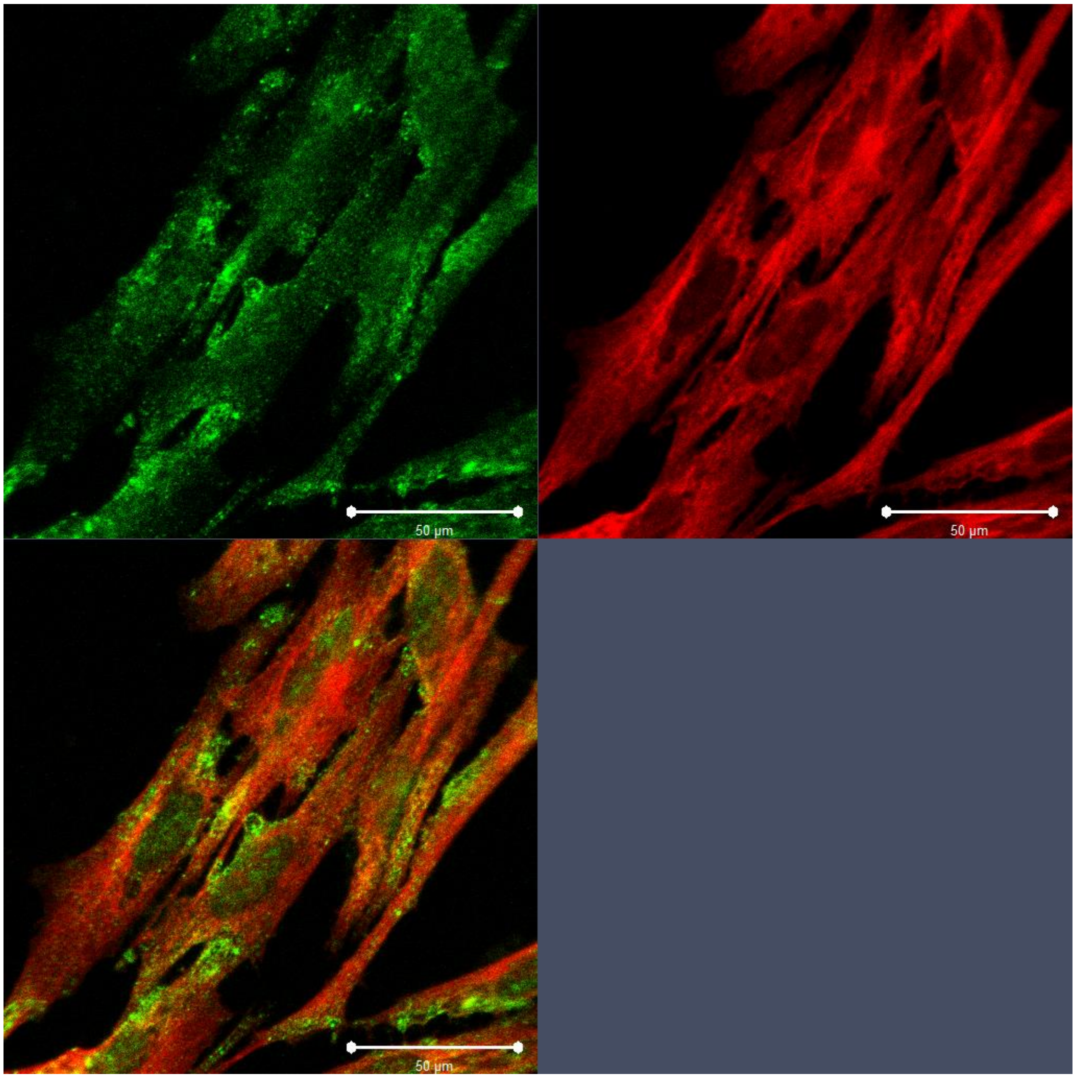

X40-NTP-3

Figure 6.

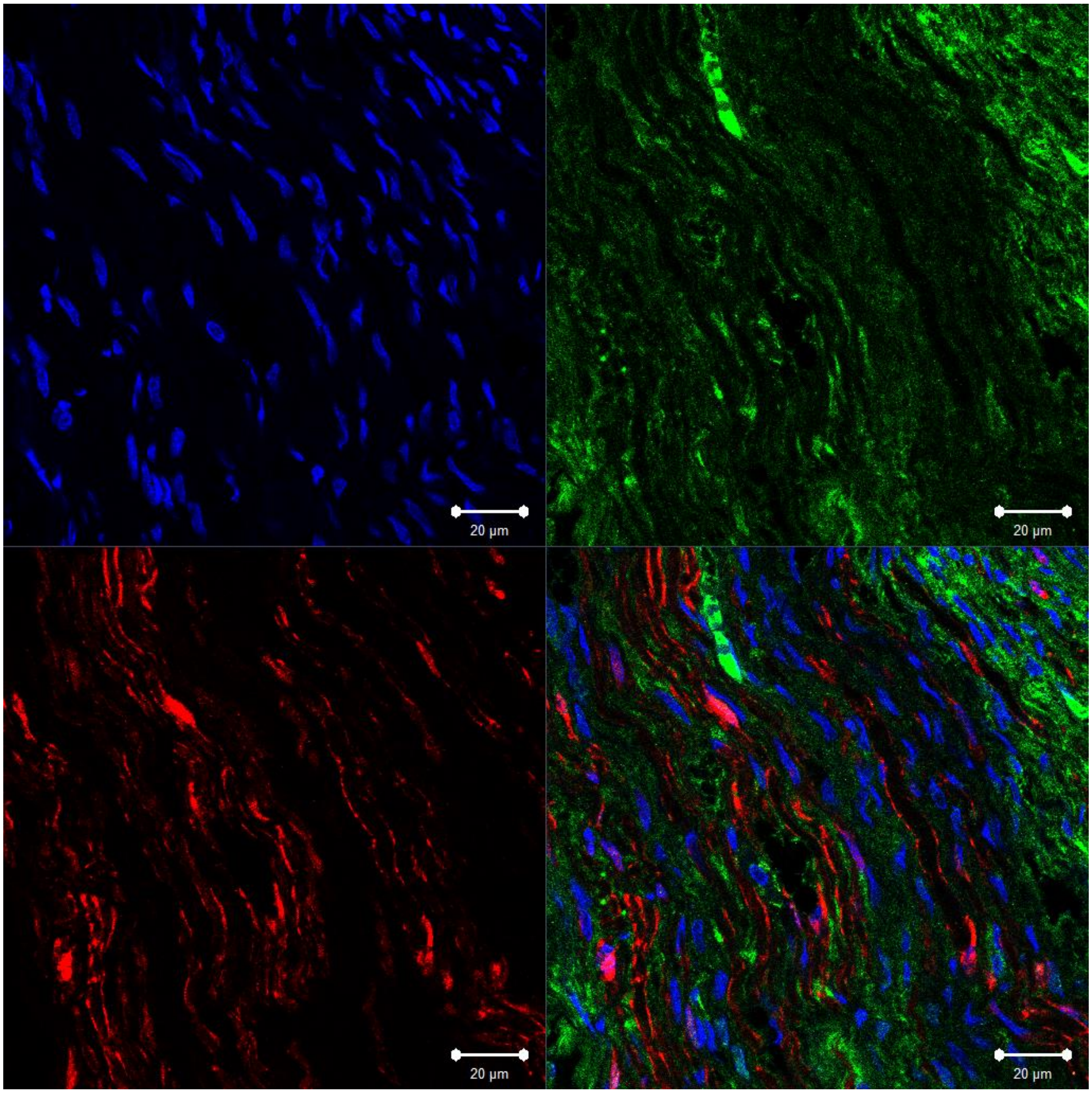

ND(TAU,S100)

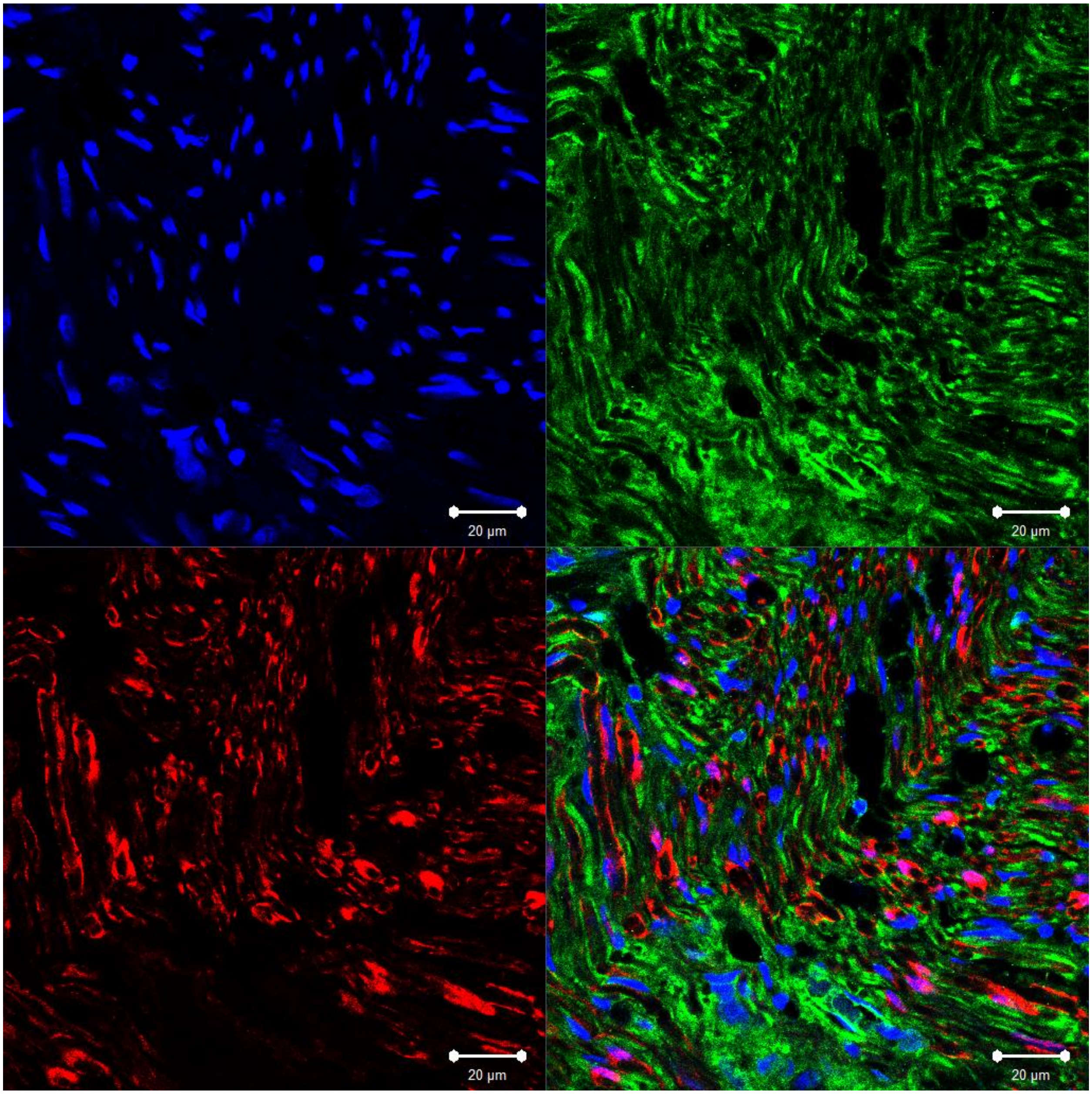

ND-NTP(TAU,S100)

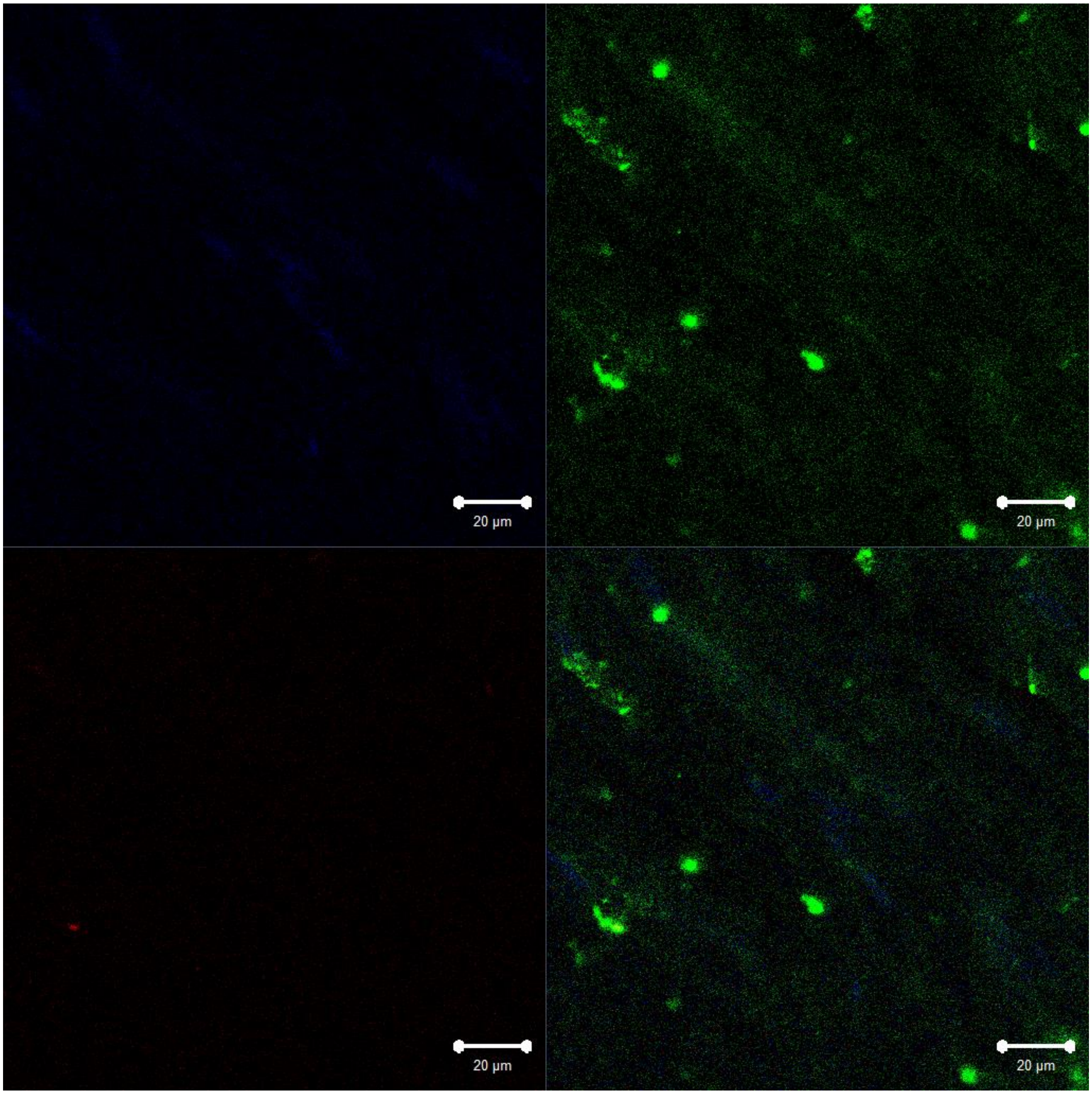

Non-ND(GAP43,MAP2)

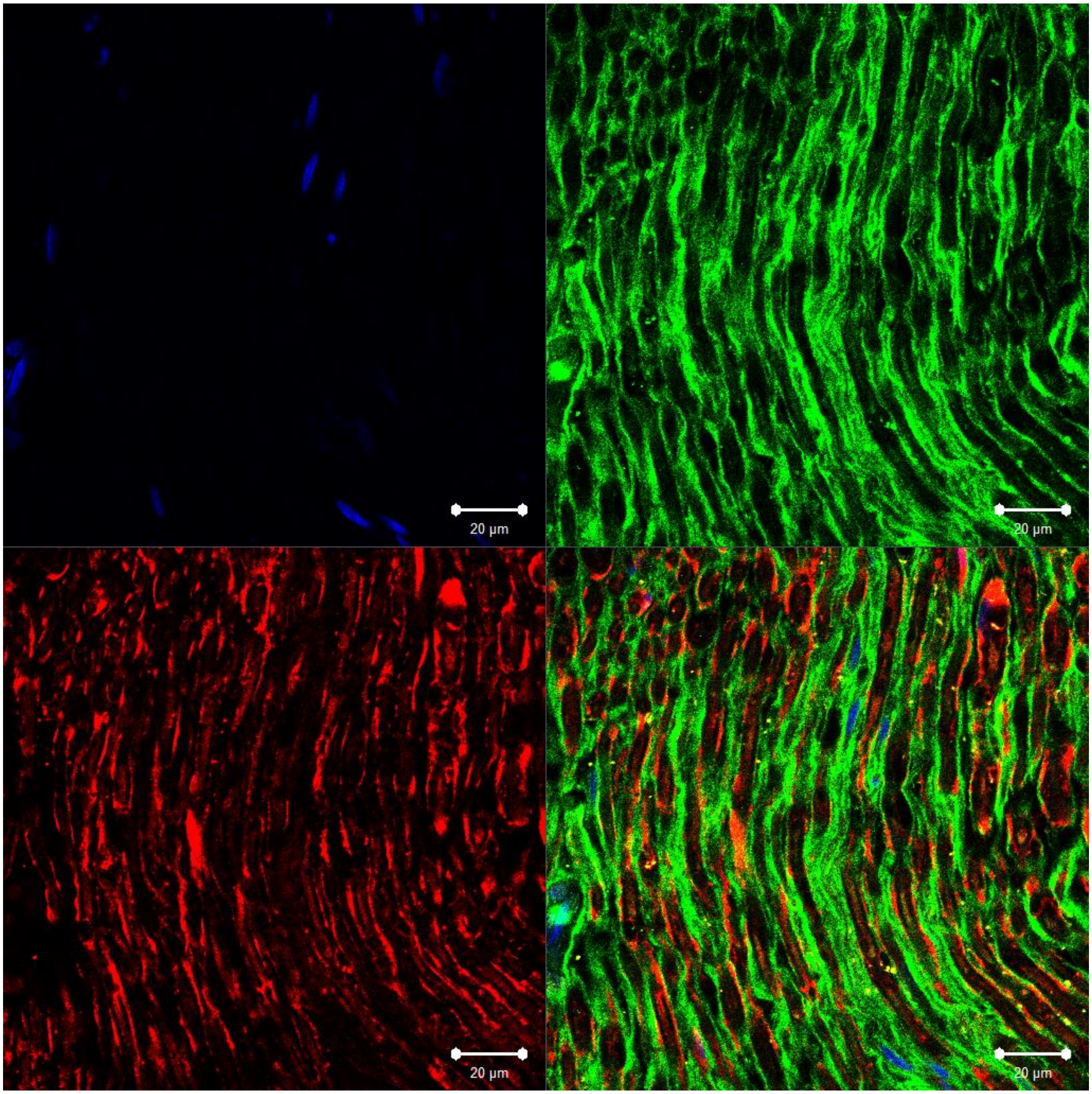

Non-ND(TAU,S100)

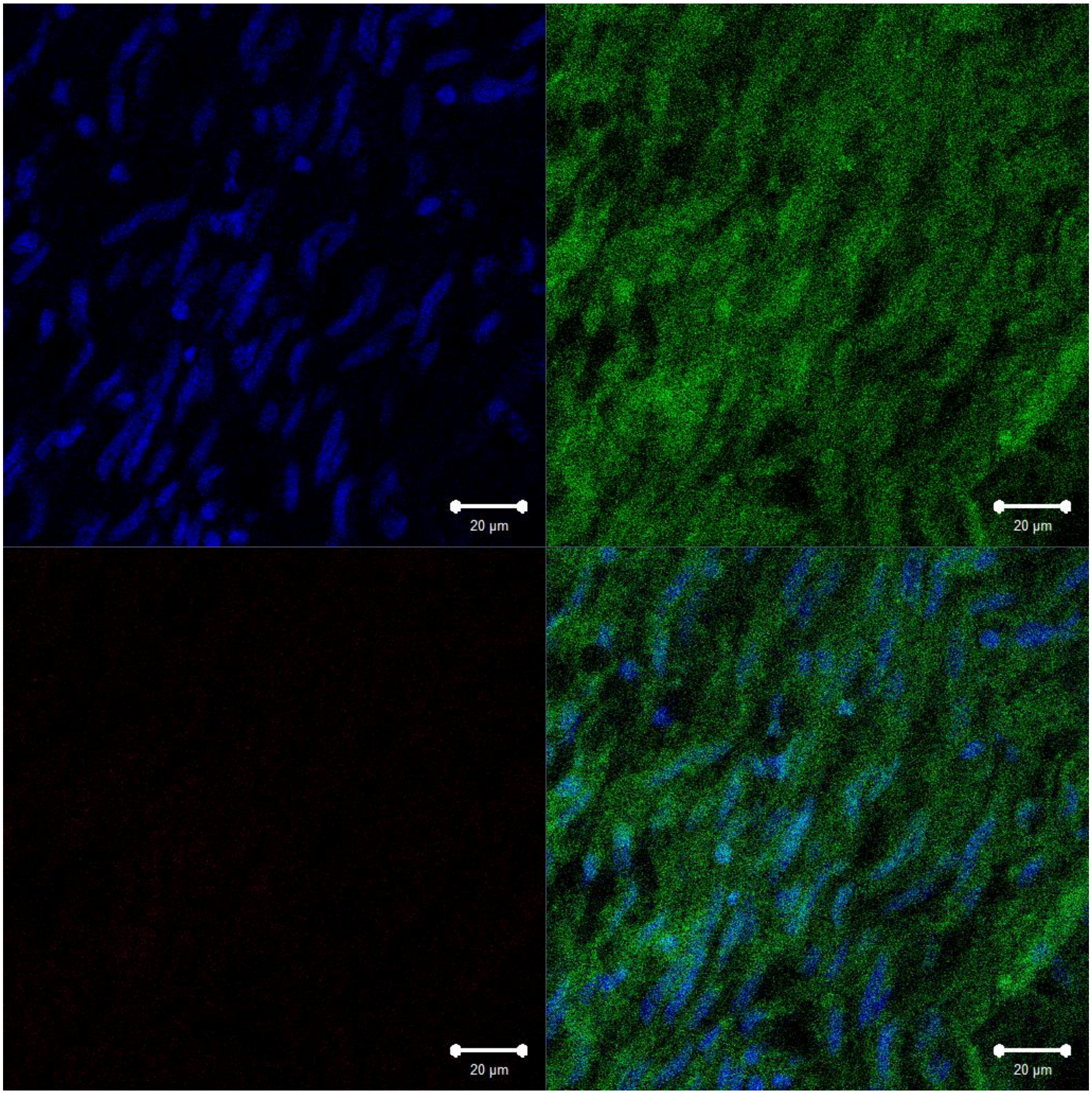

X400_ND(GAP43,MAP2)

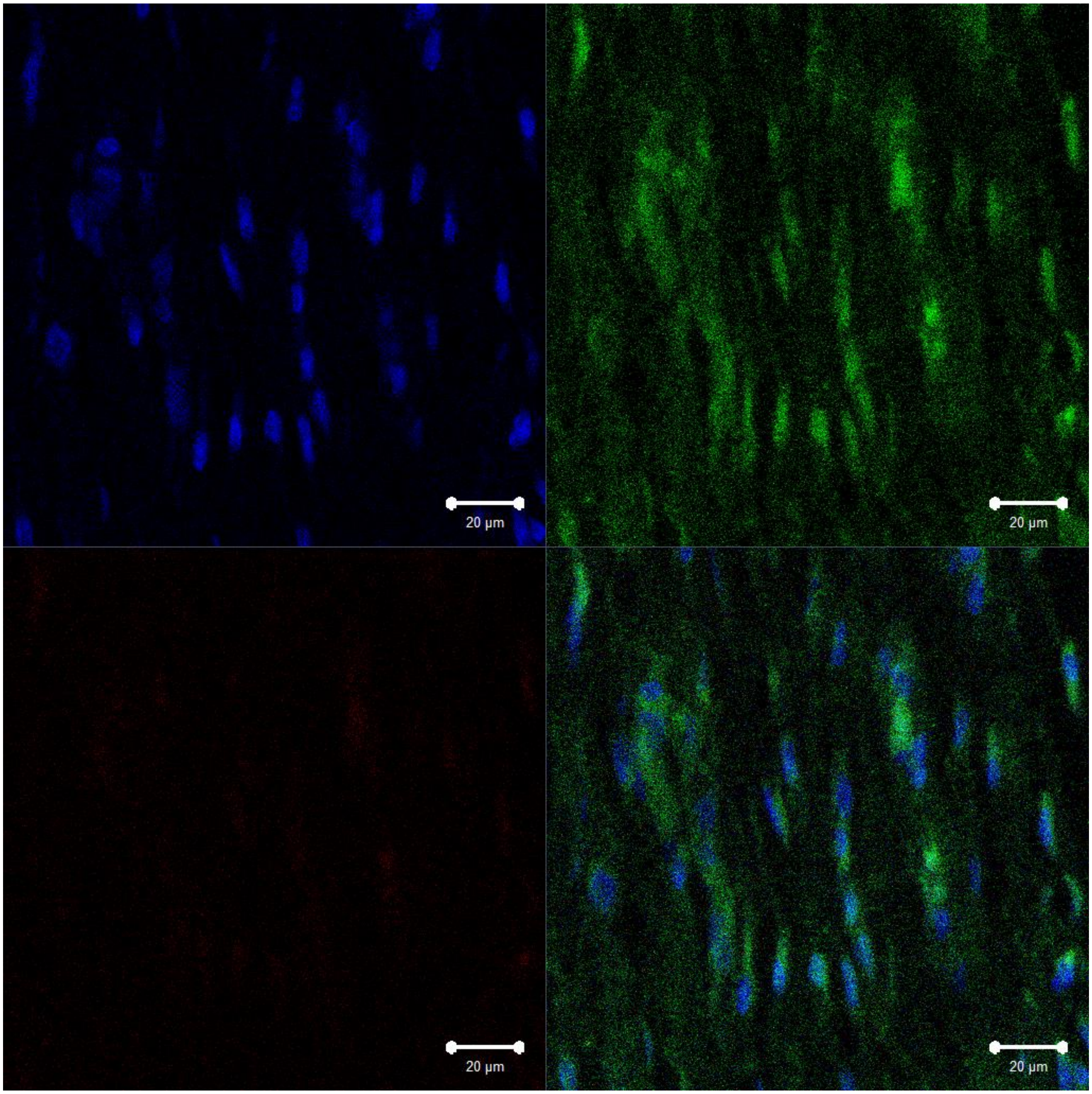

X400_ND+NTP(GAP43,MAP2)
