# Supplementary material for: Non-thermal plasma directly accelerates neuronal proliferation by stimulating axon formation
Source: Sci Rep. 2022 Sep 23;12:15868. doi: 10.1038/s41598-022-20063-4 (PMC9508269; doi:10.1038/s41598-022-20063-4)

This is the original western blot data.

The original, marker, and original + marker files of each antibody are mentioned respectively.

As it is not possible to use 1 antibody per blot, each of them were cut to fit the anti-body and used in the experiment. The reason behind it was that, the amount of the sample was not sufficient. In addition to this, the amount of anti-body was also limited.

All data is original without any changes or Photoshop.

1. GAP43(UPPER BAND)

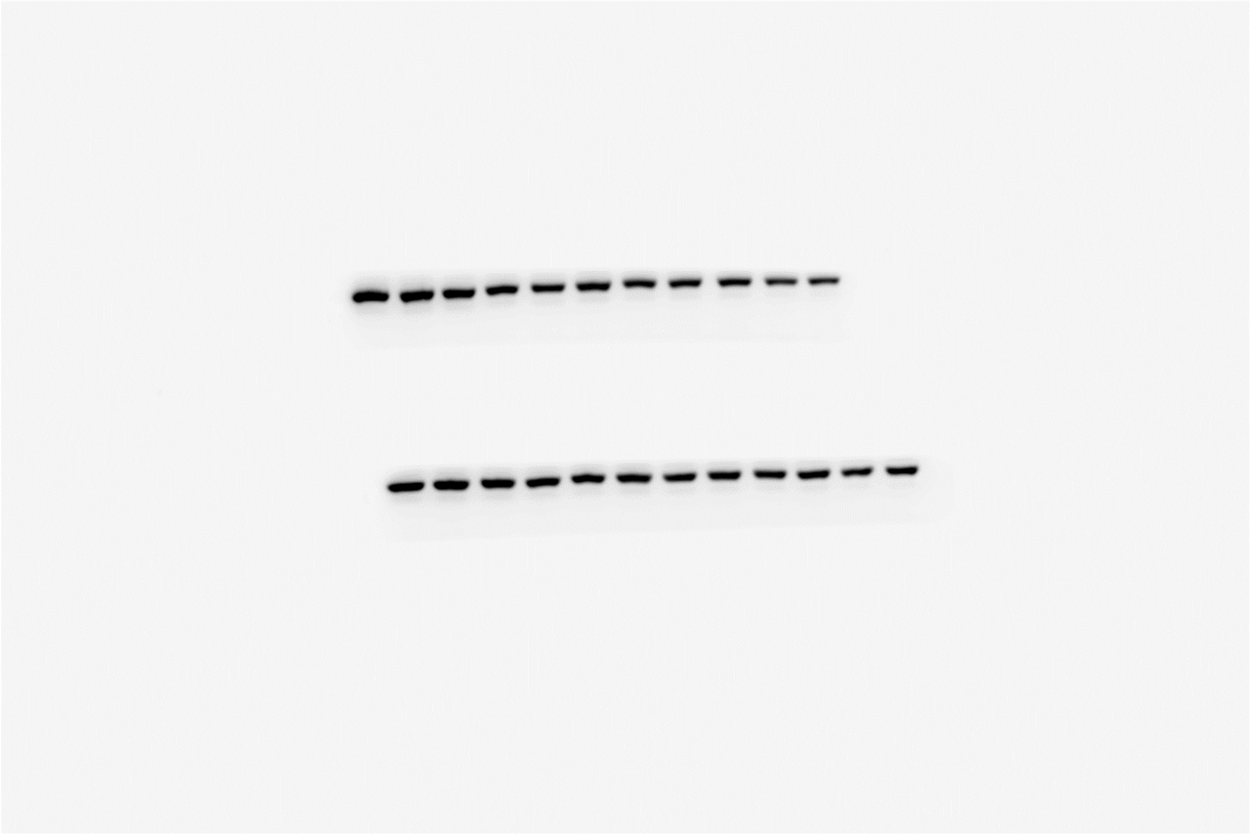

2. GAP43-15 sec- marker

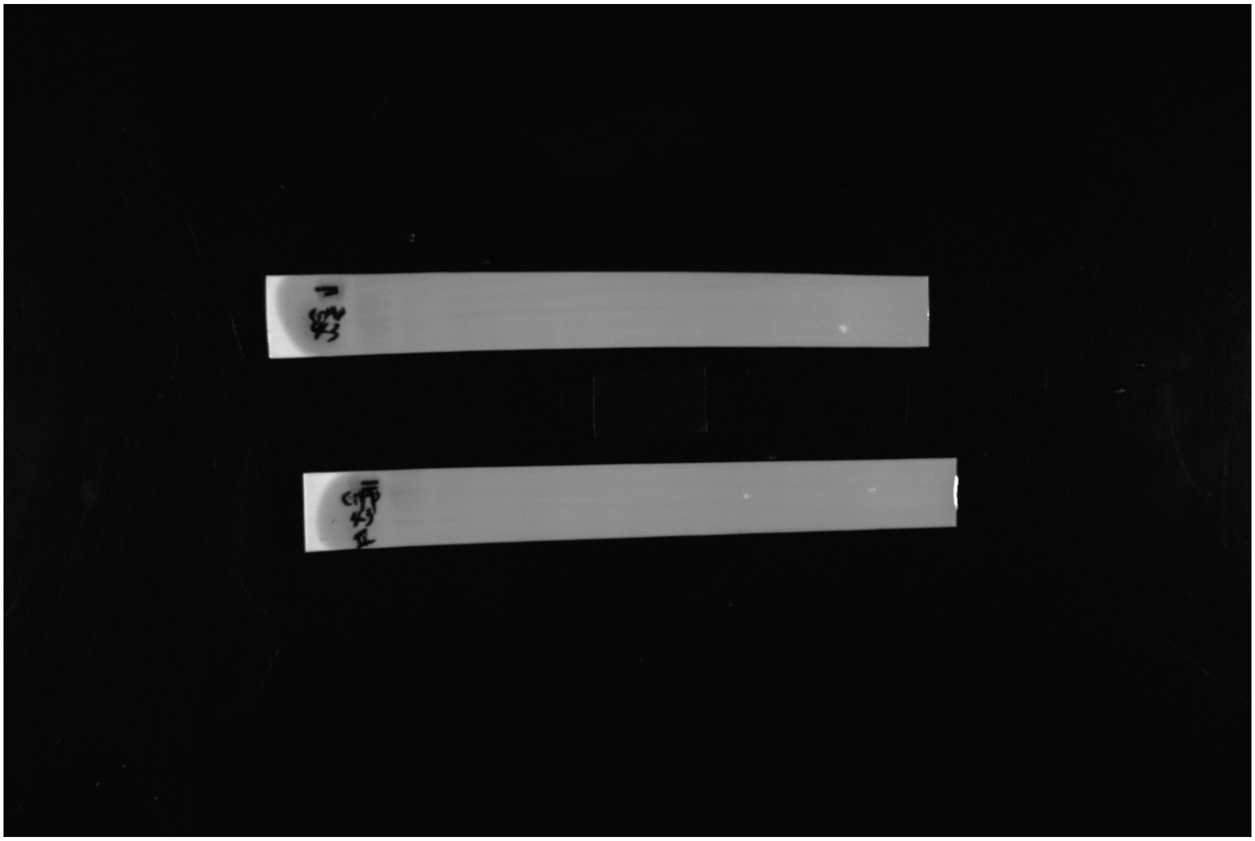

3. GAP43-marker-merge

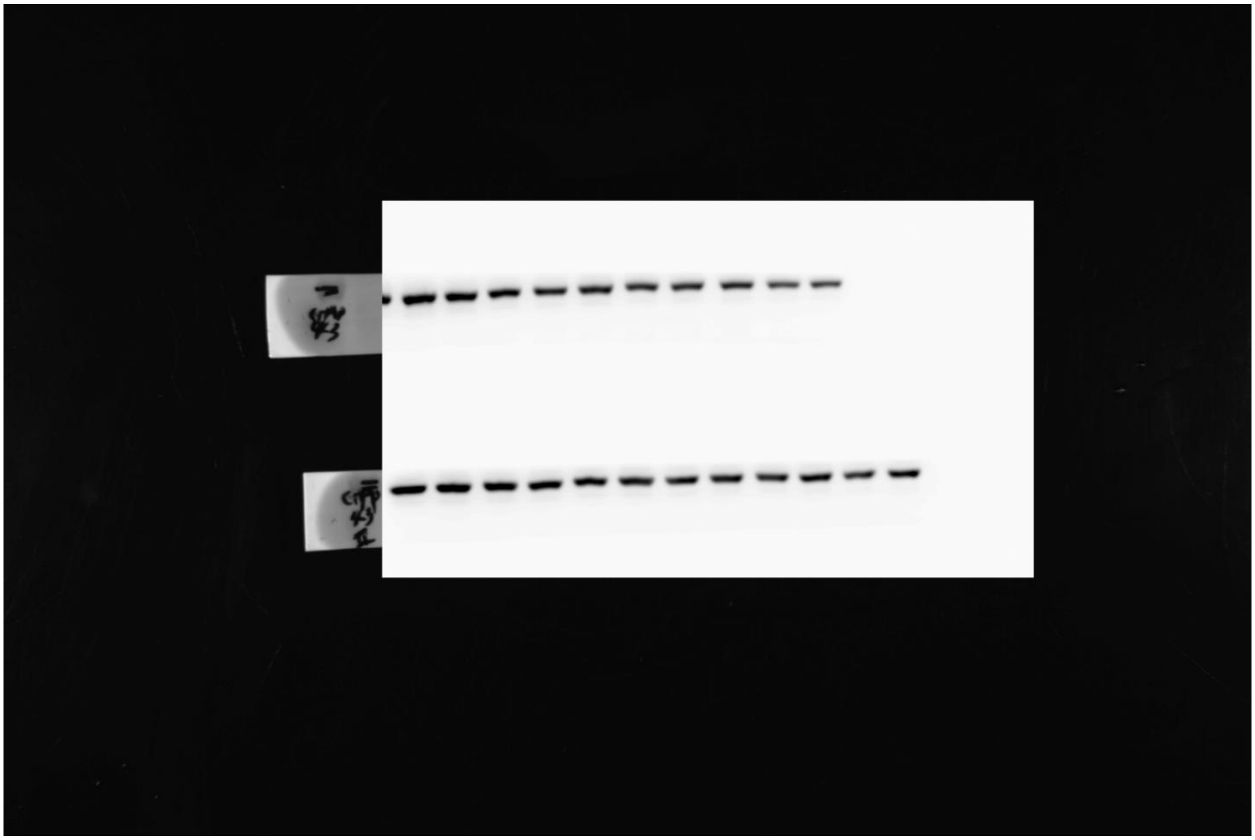

4. GAPDH

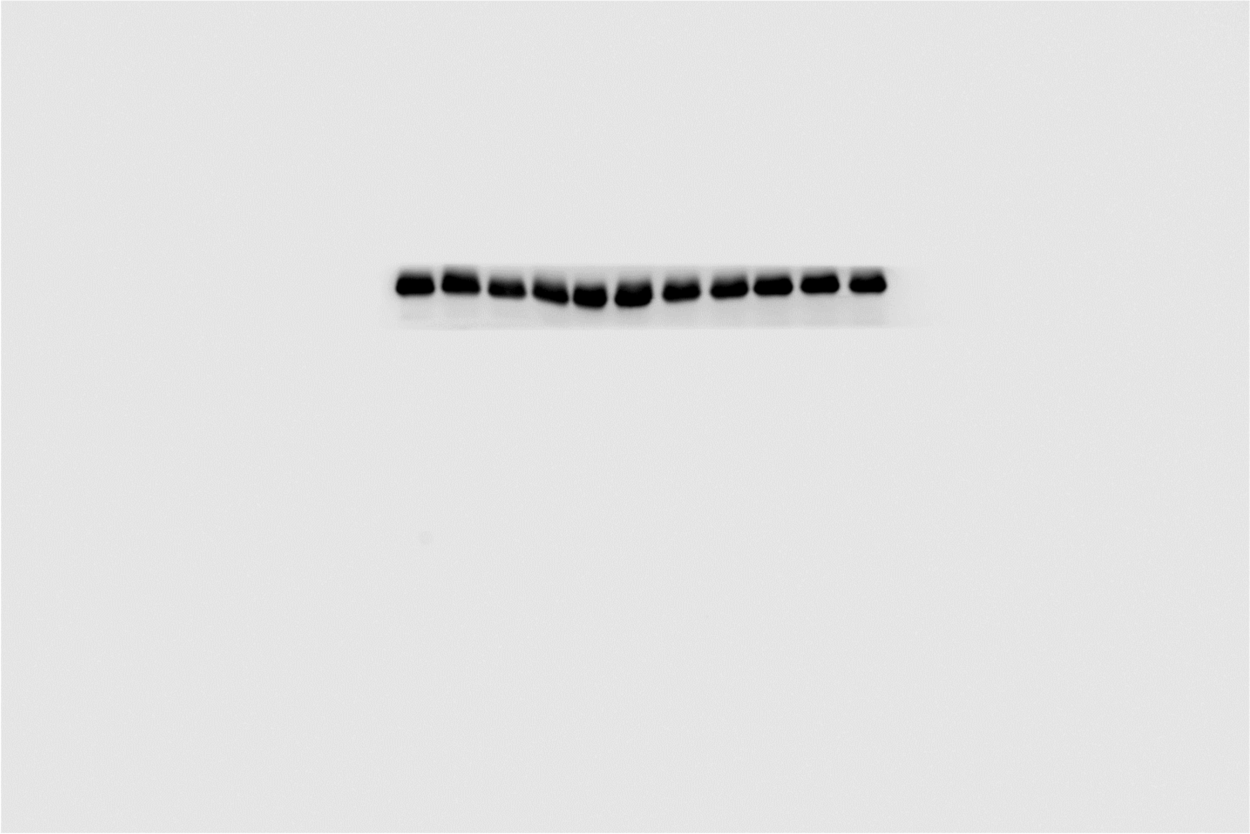

5. GAPDH- marker

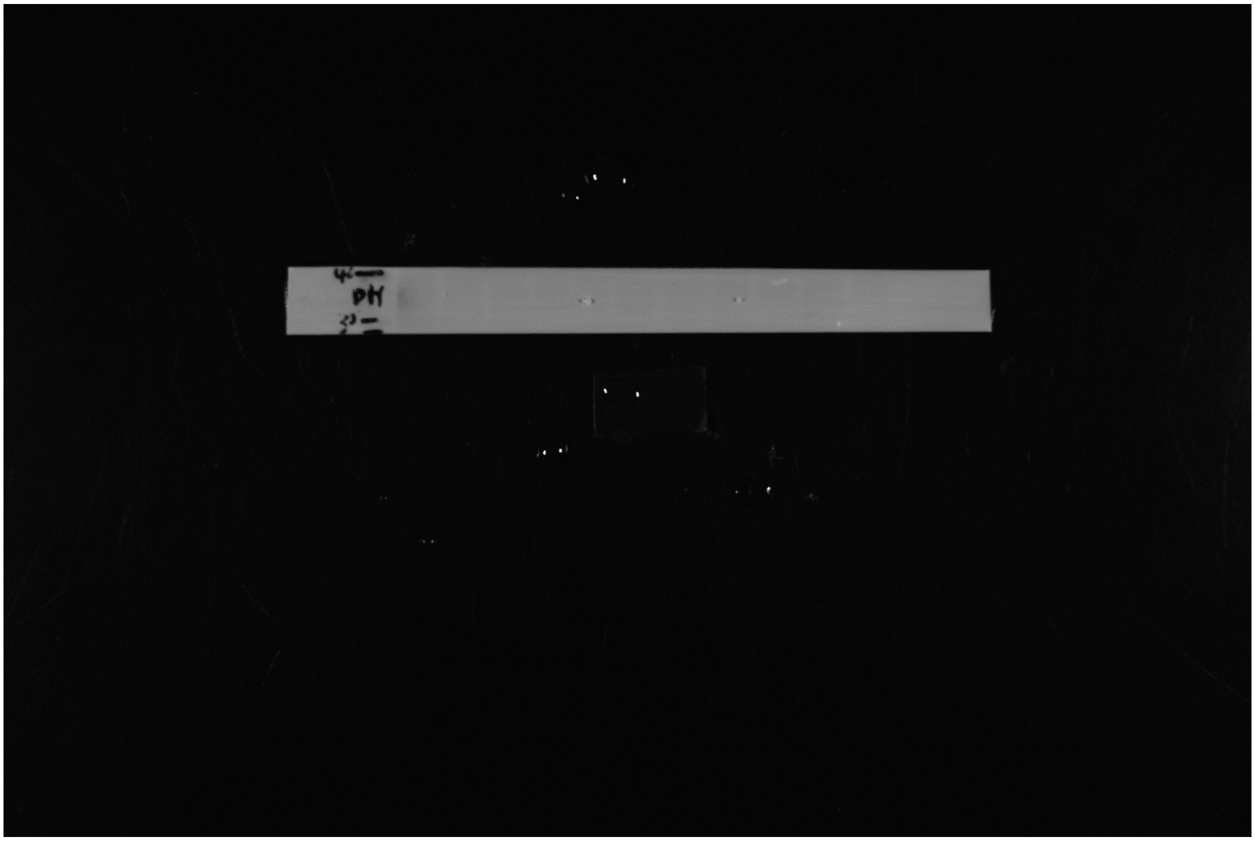

6. MAP2(upper band)

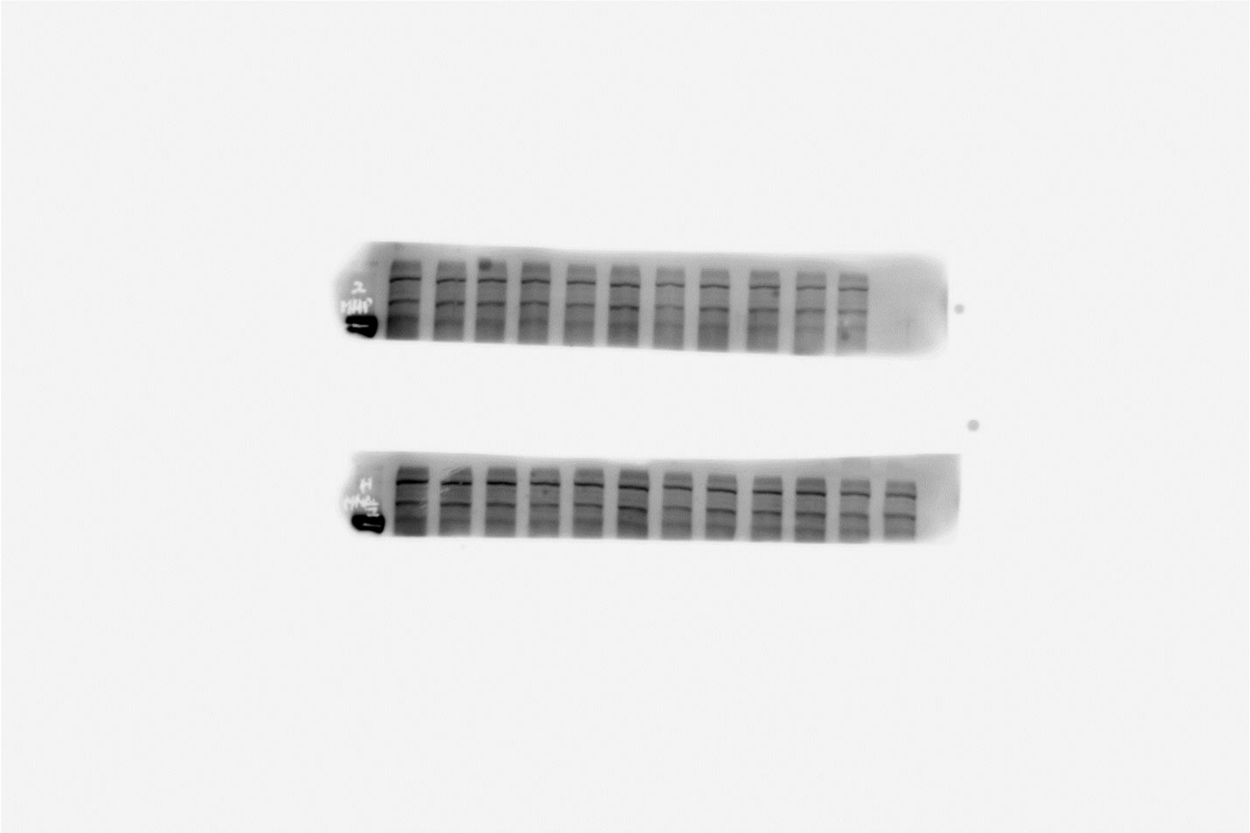

7. MAP2- marker

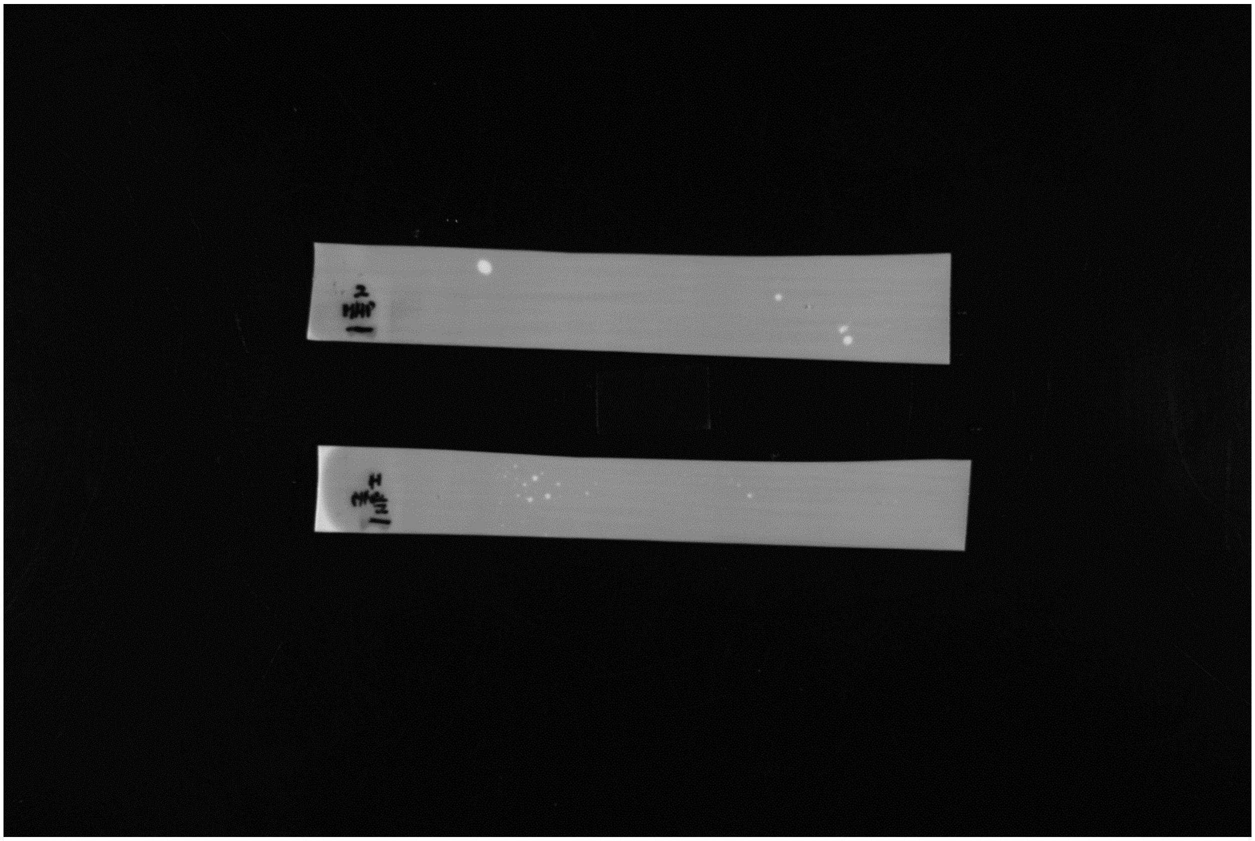

8. MAP2- marker- merge

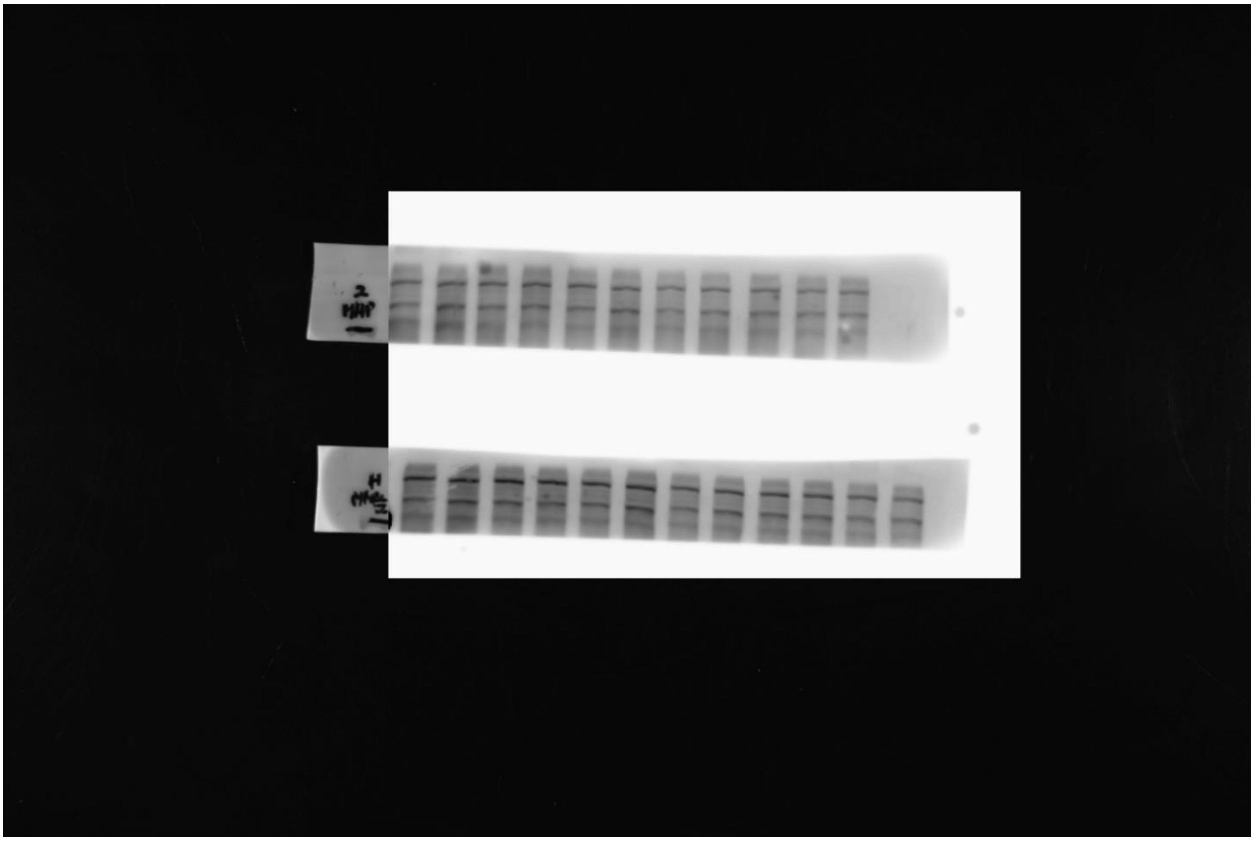

9. TAU

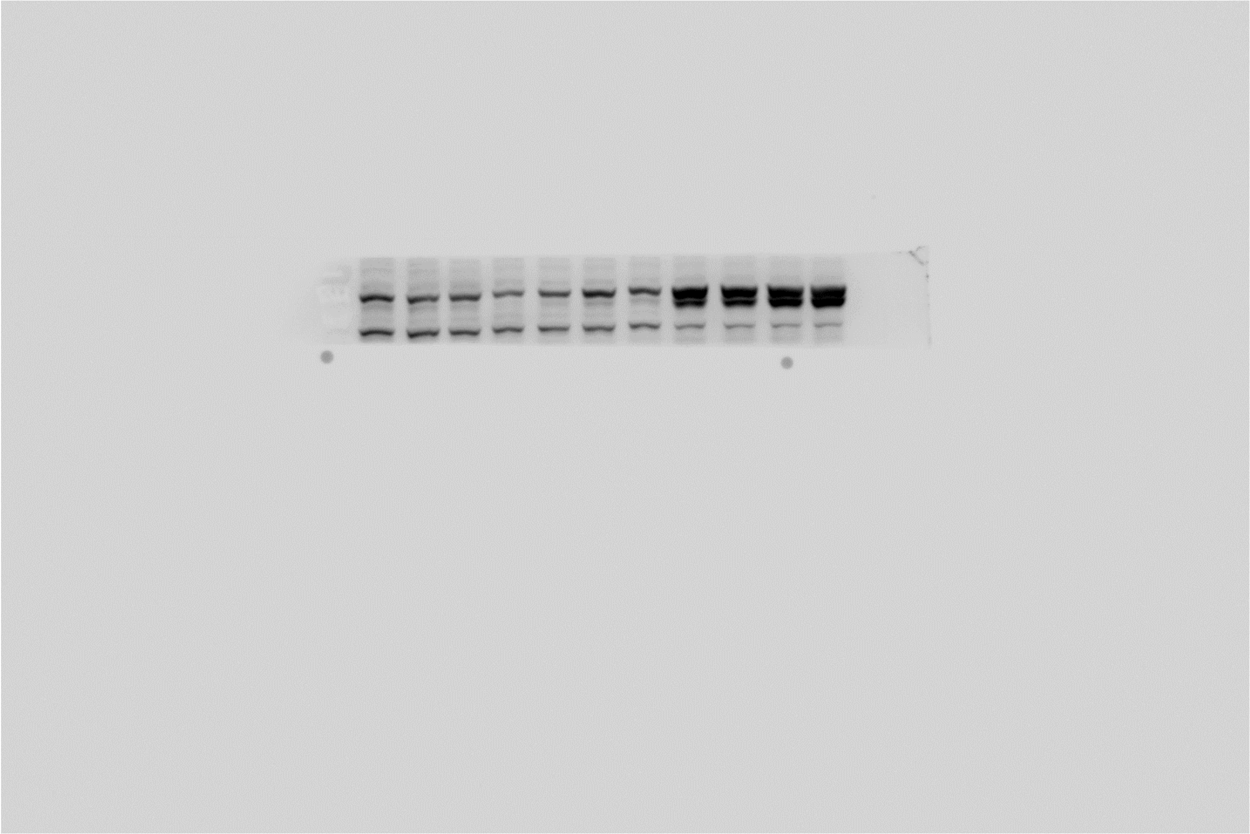

10. TAU- marker

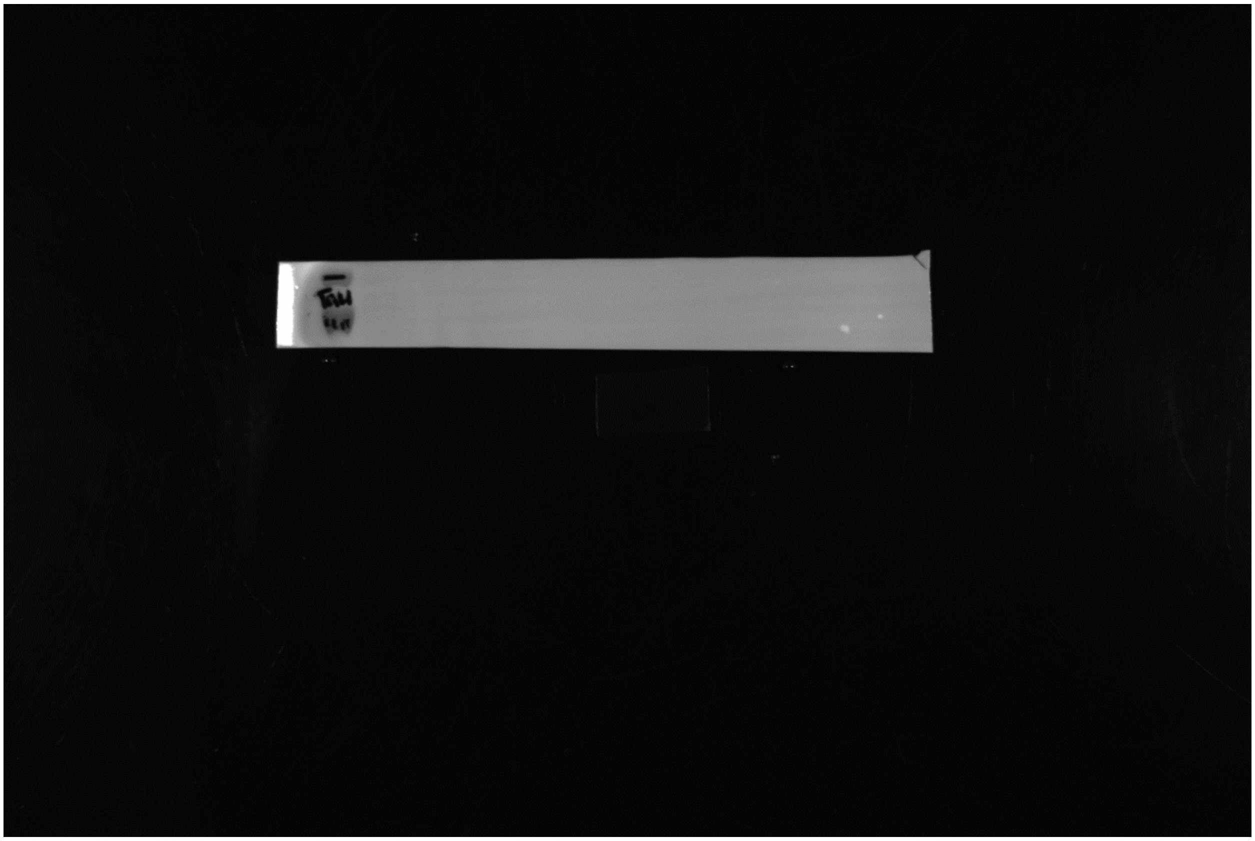

11. TAU- marker -merge

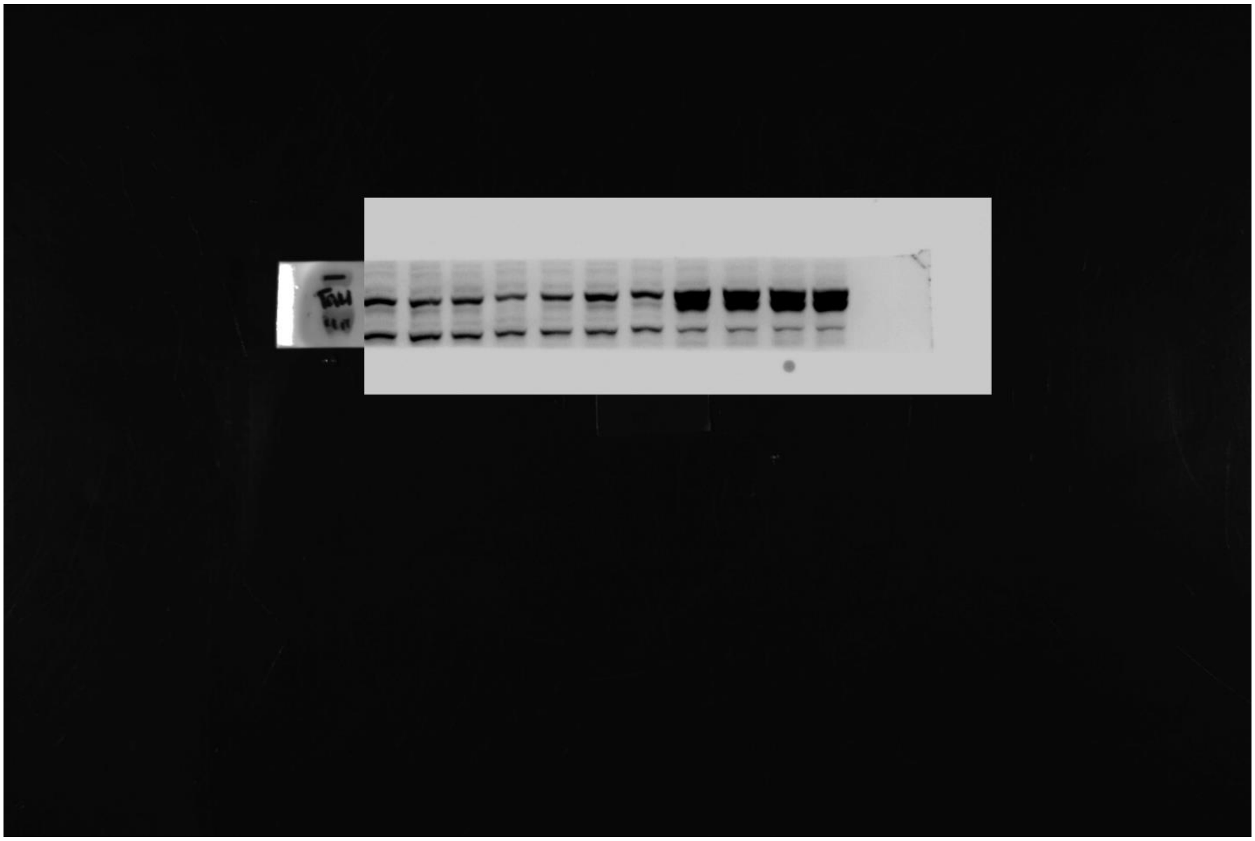

Supplement: Supplementary file 4 — Supplementary Information 4. [file 41598_2022_20063_MOESM4_ESM.pdf]
